# Using machine learning to predict COVID-19 infection and severity risk among 4510 aged adults: a UK Biobank cohort study

**DOI:** 10.1038/s41598-022-07307-z

**Published:** 2022-05-11

**Authors:** Auriel A. Willette, Sara A. Willette, Qian Wang, Colleen Pappas, Brandon S. Klinedinst, Scott Le, Brittany Larsen, Amy Pollpeter, Tianqi Li, Jonathan P. Mochel, Karin Allenspach, Nicole Brenner, Tim Waterboer

**Affiliations:** 1 Department of Food Science and Human Nutrition, Iowa State University, 2302 Osborn Drive, Ames, IA 50011-1078 USA; 2 Department of Neurology, University of Iowa, Iowa City, IA USA; 3 Iowa COVID-19 Tracker Inc., Ames, IA USA; 4 Department of Biomedical Sciences, Iowa State University, Ames, IA USA; 5 Department of Veterinary Clinical Sciences, Iowa State University, Ames, IA USA; 6 Infections and Cancer Epidemiology Division, German Cancer Research Center (DKFZ), Heidelberg, Germany

**Keywords:** Viral infection, Predictive medicine, Immunology, Biomarkers, Diseases, Risk factors

## Abstract

Many risk factors have emerged for novel 2019 coronavirus disease (COVID-19). It is relatively unknown how these factors collectively predict COVID-19 infection risk, as well as risk for a severe infection (i.e., hospitalization). Among aged adults (69.3 ± 8.6 years) in UK Biobank, COVID-19 data was downloaded for 4510 participants with 7539 test cases. We downloaded baseline data from 10 to 14 years ago, including demographics, biochemistry, body mass, and other factors, as well as antibody titers for 20 common to rare infectious diseases in a subset of 80 participants with 124 test cases. Permutation-based linear discriminant analysis was used to predict COVID-19 risk and hospitalization risk. Probability and threshold metrics included receiver operating characteristic curves to derive area under the curve (AUC), specificity, sensitivity, and quadratic mean. Model predictions using the full cohort were marginal. The “best-fit” model for predicting COVID-19 risk was found in the subset of participants with antibody titers, which achieved excellent discrimination (AUC 0.969, 95% CI 0.934–1.000). Factors included age, immune markers, lipids, and serology titers to common pathogens like human cytomegalovirus. The hospitalization “best-fit” model was more modest (AUC 0.803, 95% CI 0.663–0.943) and included only serology titers, again in the subset group. Accurate risk profiles can be created using standard self-report and biomedical data collected in public health and medical settings. It is also worthwhile to further investigate if prior host immunity predicts current host immunity to COVID-19.

## Introduction

Coronavirus disease 2019 (COVID-19), caused by a novel beta-coronavirus called severe acute respiratory syndrome coronavirus 2 (SARS-CoV-2)^1^, is a worldwide pandemic that continues to severely disrupt the economic, social, and psychological well-being of countless people. Clinical presentation of COVID-19 widely varies, ranging from asymptomatic profiles to mild symptoms like high fever or cough to acute respiratory disease syndrome and death. Given this heterogeneous symptom presentation, as well as difficulties with serology testing, vaccine administration, and the rise of variants of concern, it remains important to isolate or maximize safety for adults most at risk for COVID-19 infection and severe disease.

By extension, a large body of research has investigated potential factors that increase COVID-19 infection and disease severity risk. It is well known, for example, that adults aged > 65 years are much more likely to be hospitalized or die due to COVID-19. Obesity itself and adverse health behaviors like smoking also increase infection risk and likelihood of hospitalization^2,3^. Several age and obesity-related conditions such as cardiovascular disease, cardiometabolic diseases (e.g., type 2 diabetes), hypertension, and other disease states and syndromes are also of concern^4^. Non-white ethnicity, particularly being black regardless of country of origin, socioeconomic deprivation, and low levels of education even after adjustment for health factors point to less privilege unfortunately conferring risk^5^. Among biological markers, COVID-19 infection or severity has been related to higher C-Reactive Protein and more circulating white blood cells and lower counts of lymphocytes or granulocytes (e.g., monocytes)^6–8^. SARS-CoV-1 has a similar profile except for a relatively normal total white blood cell count^9^.

These studies are invaluable for establishing or validating risk factors to guide clinical decisions and policymaker choices. However, we ultimately need to develop risk profiles derived from these factors to accurately predict who will and will not develop COVID-19, and if a COVID-19 disease course will be mild or presumptively severe (i.e., require hospitalization). Data-driven modelling using machine learning can be used to create robust prediction models based on routinely collected biomedical data like demographics, a complete blood count, and standard medical biochemistry data. Critically, by using non COVID-19 serological data, we may gain insight into the host’s ability to fight COVID-19 by examining antibody titers that detail the host response to past infectious pathogens. This “virome” may affect host innate and adaptive immunity^9,10^. For example, human cytomegalovirus vastly changes the composition of T and B cells^11^, and may induce immune senescence that could account for worse SARS-CoV-2 infection outcomes.

Therefore, our objective was to use classification machine learning to determine how baseline measures, collected 10–14 years ago, could best predict which older adults developed COVID-19. Our second objective was to make similar predictions but for determining if someone positive with COVID-19 had a mild or severe infection. In summary, we achieved > 90% sensitivity and specificity with outstanding diagnostic value (AUC 0.969) for correctly predicting COVID-19 infection based on factors like age, biochemistry and white blood cell markers, and antibody titers to common pathogens like human cytomegalovirus, human herpesvirus 6, and chlamydia trachomatis. For COVID-19 severity, only antibody titers loaded for final models that more modestly predicted severe disease (AUC 0.803; specificity = 61.1%, sensitivity = 85.7%). Nonetheless, this report shows that trait-like baseline data from 10 to 14 years ago can better characterize who is most at risk for COVID-19 and if they are likely to be hospitalized with a presumptively severe infection. In addition, our results suggest that past infection history and antibody response may be an invaluable, novel predictor of host immunity to COVID-19 that warrants further study.

## Methods

### Study design and participants

This retrospective study involved the UK Biobank cohort^12^. UK Biobank consists of approximately 500,000 people now aged 50 to 84 years (mean age = 69.4 years). Baseline data was collected in 2006–2010 at 22 centers across the United Kingdom^13,14^. Summary data are listed in Table 1. This research involved deidentified epidemiological data. All UK Biobank participants gave written, informed consent. Ethics approval for the UK Biobank study was obtained from the National Health Service Health Research Authority North West—Haydock Research Ethics Committee (16/NW/0274), in accordance with relevant guidelines and regulations from the Declarations of Helsinki. All analyses were conducted in line with UK Biobank requirements.

**Table 1:**
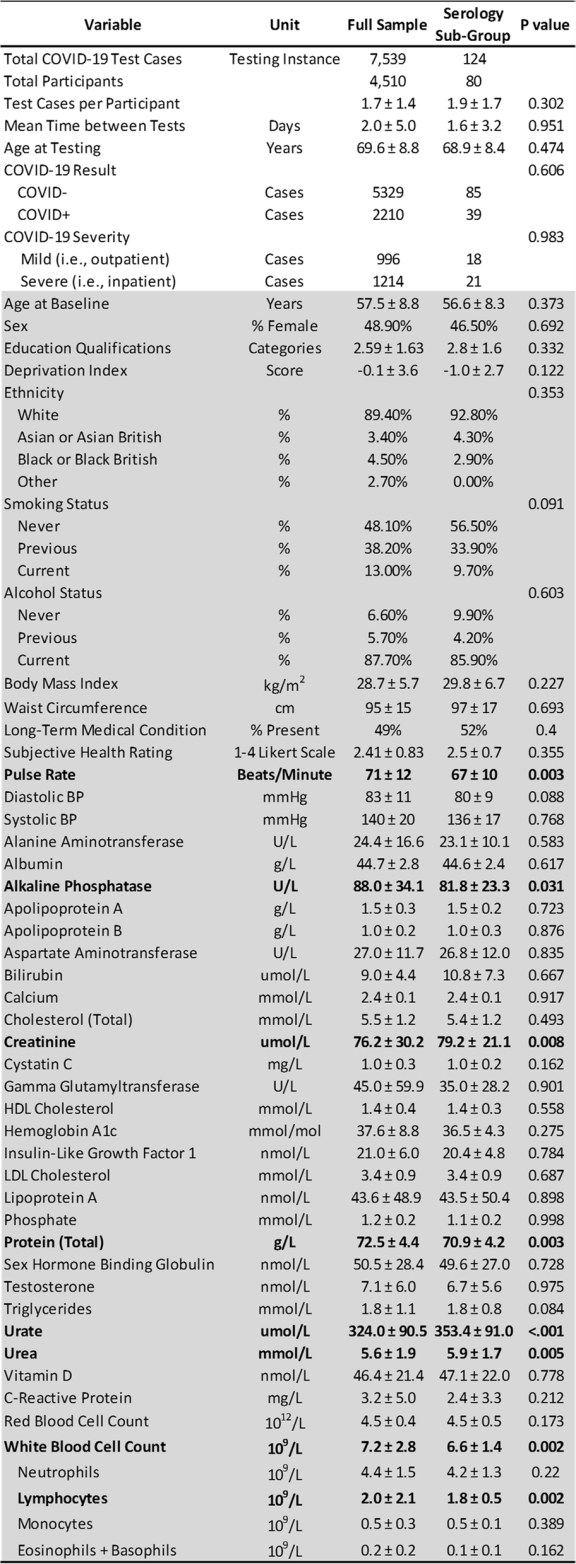
Baseline Demographics and Data Characteristics. Blood pressure (BP); high-density lipoprotein (HDL); low-density lipoprotein (LDL). A summary and comparison of data among either all participant test cases or a sub-group of test cases that also had non COVID-19 serology. All retrospective baseline data has italics. Values are in mean ± SD, percentages, or frequency. P values less than 0.05 were considered significant and applicable predictors and indices are bolded.

The following categories of predictors were downloaded: (1) demographics; (2) health behaviors and long-term disability or illness status; (3) anthropometric and bioimpedance measures of fat, muscle, or water content; (4) pulse and blood pressure; (5) a serum panel of thirty biochemistry markers commonly collected in a clinic or hospital setting; and (6) a complete blood count with a manual differential.

### Demographics

These factors included participant age in years at baseline, sex, education qualifications, ethnicity, and Townsend Deprivation Index. Sex was coded as 0 for female and 1 for male. For education, higher scores roughly correspond to progressively more skilled trade/vocational or academic training. Ethnicity was coded as UK citizens who identified as White, Black/Black British, or Asian/Asian British. The Townsend index^15^ is a standardized score indicating relative degree of deprivation or poverty based on permanent address.

### Health behaviors and conditions

This category consisted of self-reported alcohol status, smoking status, a subjective health rating on a 1–4 Likert scale (“Excellent” to “Poor”), and whether the participant had a self-described long-term medical condition. As noted in Table 1, 48.4% of participants indicated having such an ailment. We independently confirmed self-reported data with ICD-10 codes while at hospital. These conditions included all-cause dementia and other neurological disorders, various cancers, major depressive disorder, cardiovascular or cerebrovascular diseases and events, cardiometabolic diseases (e.g., type 2 diabetes), renal and pulmonary diseases, and other so-called pre-existing conditions.

### Vital signs

The first automated reading of pulse, diastolic and systolic blood pressure at the baseline visit were used.

### Body morphometrics and compartment mass

Anthropometric measures of adiposity (Body Mass Index, waist circumference) were derived as described^16^. Data also included bioelectrical impedance metrics that estimate central body cavity (i.e., trunk) and whole body fat mass, fat-free muscle mass, or water content^17^.

### Blood biochemistry and immunology

Serum biomarkers were assayed from baseline samples as described^18^. Briefly, using immunoassay or clinical chemistry devices, spectrophotometry was used to initially quantify values for 34 biochemistry analytes. UK Biobank deemed 30 of these markers to be suitably robust. We rejected a further 4 markers due data missingness > 70% (estradiol, rheumatoid factor), or because there was strong overlap with multicollinear variables that had more stable distributions or trait-like qualities (glucose rejected vs. glycated hemoglobin/hba1c; direct bilirubin rejected vs. total bilirubin). A complete blood count with a manual differential was separately processed for red and white blood cell counts, as well as white cell sub-types.

### Serology measures for non COVID-19 infectious diseases

As described (http://biobank.ctsu.ox.ac.uk/crystal/crystal/docs/infdisease.pdf), among 9695 randomized UK Biobank participants selected from the full 500,000 participant cohort, baseline serum was thawed and pathogen-specific assays run in parallel using flow cytometry on a Luminex bead platform^19^.

Here, the goal of the multiplex serology panel was to measure multiple antibodies against several antigens for different pathogens, reducing noise and estimating the prevalence of prior infection and seroconversion in at least UK Biobank. All measures were initially confirmed in serum samples using gold-standard assays with median sensitivity and specificity of 97.0% and 93.7%, respectively. Antibody load for each pathogen-specific antigen was quantified using median fluorescence intensity (MFI). Because seropositivity is difficult to assess for several pathogens, we did not use pathogen prevalence as a predictor in models.

Table 2 shows the selected pathogens, their respective antigens, estimated prevalence of each pathogen based roughly on antibody titers, and assay values. This array ranges from delta-type retroviruses like human T-cell lymphotropic virus 1 that are rare (< 1%) to human herpesviruses 6 and 7 that have an estimated prevalence of more than 90%.

**Table 2:**
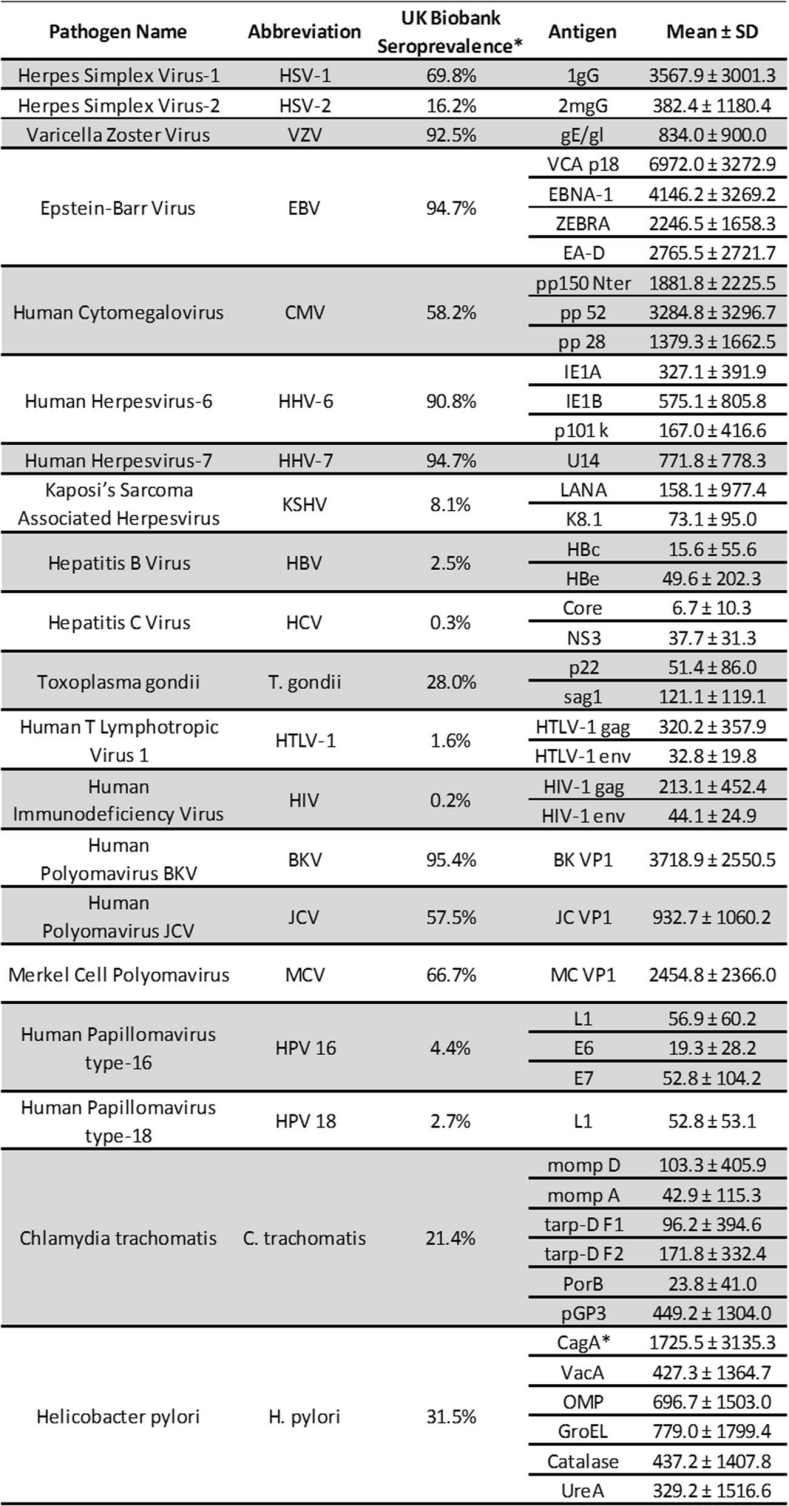
Baseline characteristics of infectious disease serology from 2006 to 2010. Antibody levels are specific to each antigen and expressed in Median Fluorescence Intensity (MFI) units. Seroprevalence of at least the main UK Biobank cohort was estimated on samples from 9695 randomized participants, as described in white papers (see “Methods”). The “bold” and “italics” shading are used to distinguish between pathogens and their respective antigens. ^a^CagA levels are based on roughly half of the original sample due to a technical lab error.

### COVID-19 testing

Our study was based on COVID PCR test data available from March 16th to May 19th 2020. Specifically, we used the May 26th, 2020 tranche of COVID-19 polymerase chain reaction (PCR) data from Public Health England. There were 4510 unique participants that had 7539 individual tests administered, hereafter called test cases. To characterize each test case, UK Biobank had a binary variable for test positivity (“result”) and a separate binary variable for test location (“origin”). For the positivity variable, a COVID-19 test was coded as negative (0) or positive (1). The second binary variable represented whether the COVID-19 test occurred through a setting that was out-patient (0) or in-patient at hospital (1). As a proxy for COVID-19 severity later verified by electronic health records and death certificates^20^, and as done in other UK Biobank reports^21^, a test case first needed to be positive for COVID-19 (i.e., the test had a ‘1’ value for the positivity variable). Next, if the positive test case occurred in an out-patient setting the infection was considered mild (i.e., 0), whereas for in-patient hospitalization it was considered severe (i.e., 1). Thus, two separate sets of analyses were run to predict: (1) COVID-19 positivity; and (2) COVID-19 severity.

### Statistical analyses

For a more technical description of the specific machine learning algorithm used to predict test case outcomes, see Supplementary Text 1. Supplementary Text 2 has an in-depth description and analysis of within-subject variation for outcome measures and number of test cases done per participant. Briefly, this variability was modest and had no significant impact on classifier model performance. SPSS 27 was used for all analyses and Alpha set at 0.05. Preliminary findings suggested that baseline serology data performed well in classifier models, despite a limited number of participants with serology. To determine if this serology sub-group was noticeably different from the full sample, Mann–Whitney U and Kruskal–Wallis tests were done (Alpha = 0.05). Hereafter, separate sets of classification analyses were performed for: (1) the full cohort; and (2) the sub-group of participants that had serology data. In other words, due to the imbalance of sample sizes and by definition the absence or presence of serology data, classifier performance in the serology sub-group was never statistically compared to the full cohort.

Next, linear discriminant analysis (LDA) was used in two separate sets of analyses to predict either: (1) COVID-19 diagnosis (negative vs. positive); or (2) COVID-19 infection severity (mild vs. severe). Again, for a given test case, COVID-19 severity would be examined only among participants who tested positive for COVID-19. LDA is a regression-like classification technique that finds the best linear combination of predictors that can maximally distinguish between groups of interest. To determine how useful a given predictor or related group of predictors (e.g., demographics) were for classification, simple forced entry models were first done. Subsequently, to derive “best fit,” robust models of the data, stepwise entry (Wilks’ Lambda, F value entry = 3.84) was used to exclude predictors that did not significantly account for unique variance in the classification model. This data reduction step is critical because LDA can lead to model overfitting when there are too many predictors relative to observations^22,23^, which are COVID-19 test cases for our purposes. Finally, because there were multiple test cases that could occur for the same participant, this would violate the assumption of independence. To guard against this problem, we used Mundry and Sommer’s permutation LDA approach. Specifically, for each LDA model, permutation testing (1000 iterations, P < 0.05) was done by randomizing participants across groupings of test cases to confirm robustness of the original model^24^.

LDA model overfitting can also occur when there is a sample size imbalance. Because there were many more negative vs. positive COVID-19 test cases in the full sample (5329 vs. 2210), the negative test group was undersampled. Specifically, a random number generator was used to discard 2500 negative test cases at random, such that the proportion of negative to positive tests was now 55% to 45% instead of 70.6 to 29.4%. Results without undersampling were similar (data not shown). No such imbalance was seen for COVID-19 severity in the full sample or for the serology sub-group. A typical holdout method of 70% and 30% was used for classifier training and then testing^25^. Finally, a two-layer non-parametric approach was used to determine model significance and estimated fit of one or more predictors. First, bootstrapping^26^ (95% Confidence Interval, 1000 iterations) was done to derive estimates robust against any violations of parametric assumptions. Next, ‘leave-one-out’ cross-validation^22^ was done with bootstrap-derived estimates to ensure that models themselves were robust. Collectively, the stepwise LDA models ensured that estimation bias of coefficients would be low because most predictors are “thrown out” before models are generated using the remaining predictors.

For each LDA classification model, outcome threshold metrics included: specificity (i.e., true negatives correctly identified), sensitivity (i.e., true positives correctly identified), and the geometric mean (i.e., how well the model predicted both true negatives and positives). The area under the curve (AUC) with a 95% confidence interval (CI) was reported to show how well a given model could distinguish between a COVID-19 negative or positive test result, and separately for COVID-19 + test cases if the disease was mild or severe. Receiver operating characteristic (ROC) curves plotted sensitivity against 1-specificity to better visualize results for sets of predictors and a final stepwise model. For stepwise models, the Wilks’ Lambda statistic and standardized coefficients are reported to see how important a given predictor was for the model. A lower Wilks’ Lambda corresponds to a stronger influence on the canonical classifier.

### Ethics declarations

Ethics approval for the UK Biobank study was obtained from the National Health Service Health Research Authority North West—Haydock Research Ethics Committee (16/NW/0274). All analyses were conducted in line with UK Biobank requirements.

## Results

As shown in Table 1, 7539 total test cases for COVID-19 were conducted among 4510 UK Biobank participants (69.6 ± 8.8 years) between March 16th to May 19th 2020, either in outpatient or inpatient settings. There were 5329 negative cases and 2210 positive cases. Of the positive cases, there were 996 mild and 1214 presumptively severe disease outcomes. Baseline data from 10 to 14 years ago (mean = 11.22 years) was available for demographic, laboratory, biochemistry, and clinical indices. Similar data from 2020 was not available. A central theme of this report is examining prediction models for the so-called full sample, but also an entirely separate set of models for a sub-group of test cases with serology data (Table 2). Table 1 indicates that the full cohort and serology sub-groups largely did not differ on most measures. A few significant differences were clinically unremarkable for the serology sub-cohort and well within the range of normal values, including lower pulse rate, several markers reflecting better kidney function, and lower total white blood cell count due to fewer lymphocytes.

Next, each baseline variable was used to predict COVID-19 infection for a given test case. For context, model performance was judged by: (1) the AUC as a measure of probability, where 0.5 is at-chance prediction and 1.0 is perfect prediction; and (2) the geometric mean or g-mean as a threshold metric, with a higher percentage corresponding to greater likelihood of correctly identifying both true positives and true negatives. Among all participants (Supplementary Table 1), as expected, model fit was poor for individual predictors that loaded significantly (mean AUC 0.532; AUC range 0.517–0.551). For example, known risk factors included larger body composition indices (AUC 0.526–0.548; g-mean = 16.2–29.6%), older age (AUC 0.522; g-mean = 38.8%), and markers of dysmetabolism like higher hba1c % (AUC 0.537; g-mean = 13.3%) and high diastolic blood pressure (AUC 0.519; g-mean = 17.9%).

For the serology sub-group (Supplementary Table 2), several established risk factors that loaded had better overall fit (mean AUC 0.656, AUC range = 0.601–0.731). Like the full sample, examples included larger body mass like fat-free mass (AUC 0.687; g-mean = 65.0%), hba1c % (AUC 0.638; g-mean = 52.8%), and diastolic blood pressure (AUC 0.633; g-mean = 55.2%). Some unexpected factors included total protein (AUC 0.662; 65.8%) and testosterone (AUC 0.731; g-mean = 55.8%). We then tested if antibody titers to antigens of 20 rare to common infectious pathogens could predict host immunity in 2020 to COVID-19. As shown in Supplementary Table 3, antibody titers to 15 antigens across 12 pathogens each performed as well on average as other non-serology predictors (mean AUC 0.653, AUC range 0.612–0.710). Specificity and sensitivity were notable for antibody levels to the pp150 Nter antigen to human cytomegalovirus (g-mean = 61.0%) and U14 to Human Herpes Virus-7 (g-mean = 66.1%), given their prevalence in the sample.

**Figure 1:**
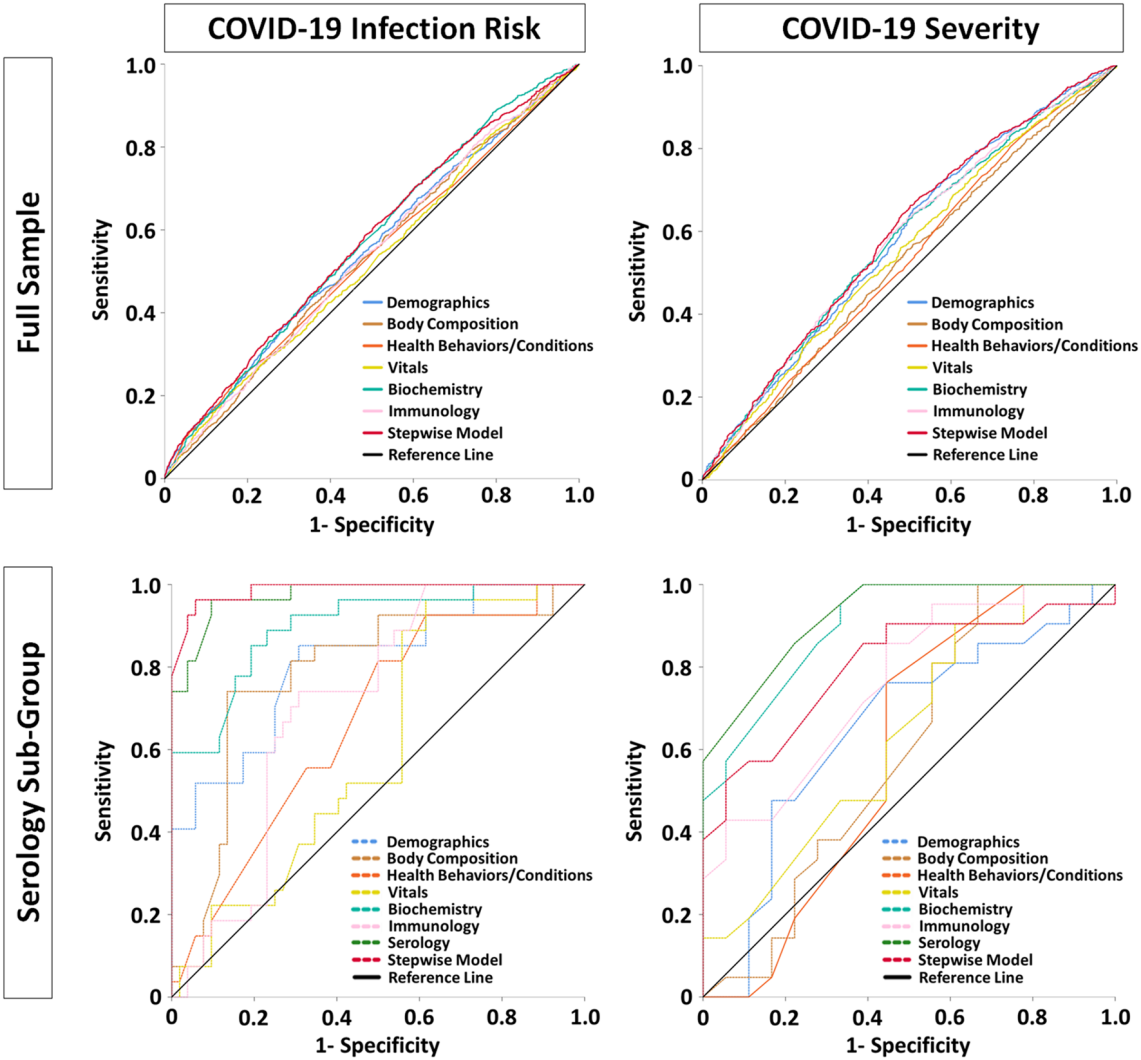
Receiver Operating Characteristics (ROC) curves illustrating the relative classifier performance of various sets of predictors. Outcomes of interest were COVID-19 infection risk and whether an infection was mild or severe. Two separate sets of analyses were done for the full tested sample and a sub-group of participants with serology data. Test statistics for predictors are provided in Tables 3 and 4.

Next, sets of similar predictors were used to gauge how well they collectively predicted COVID-19 infection, as listed in Table 3 and shown using ROC curves in Fig. 1. A stepwise model was also used to create a classifier that only included predictors where each provided unique predictive utility, and to minimize likelihood of overfitting models. For the full sample (Table 3, top row), model results are shown for each sets of predictors and the stepwise model, specifically their degree of likelihood to identify COVID-19 negative and positive test cases. The stepwise model correctly identified negative and positive test cases 83.0% and 23.8% of the time respectively. Supplementary Table 4 (top row) illustrates that the stepwise model included triglycerides, body mass, age, ethnicity, and other known risk factors for COVID-19. Importantly, for the serology sub-group (Table 3, bottom row), forced entry models showed worse performance compared to the same models among the full sample, except for the biochemistry set. This suggests that small sample size for the serology sub-group did not lead to model overfitting. While the forced entry serology model itself is likely overfitted, the stepwise model loaded 15 predictors and performed well (g-mean = 0.920). As shown in Supplementary Table 4 (bottom row), predictors that loaded in the stepwise model included antibody titers for antigens of several common pathogens (e.g., human cytomegalovirus, *C. trachomatis*), lipid markers, age, white and red cell counts, and testosterone. Due to potential concerns with model overfitting, the stepwise model was re-run with only predictors that had individually loaded significantly in forced entry models (Supplementary Tables 2 and 3). This stepwise model had 10 variables and achieved a g-mean of 85.4%, suggesting that stepwise models were not overfitted.

**Table 3:**
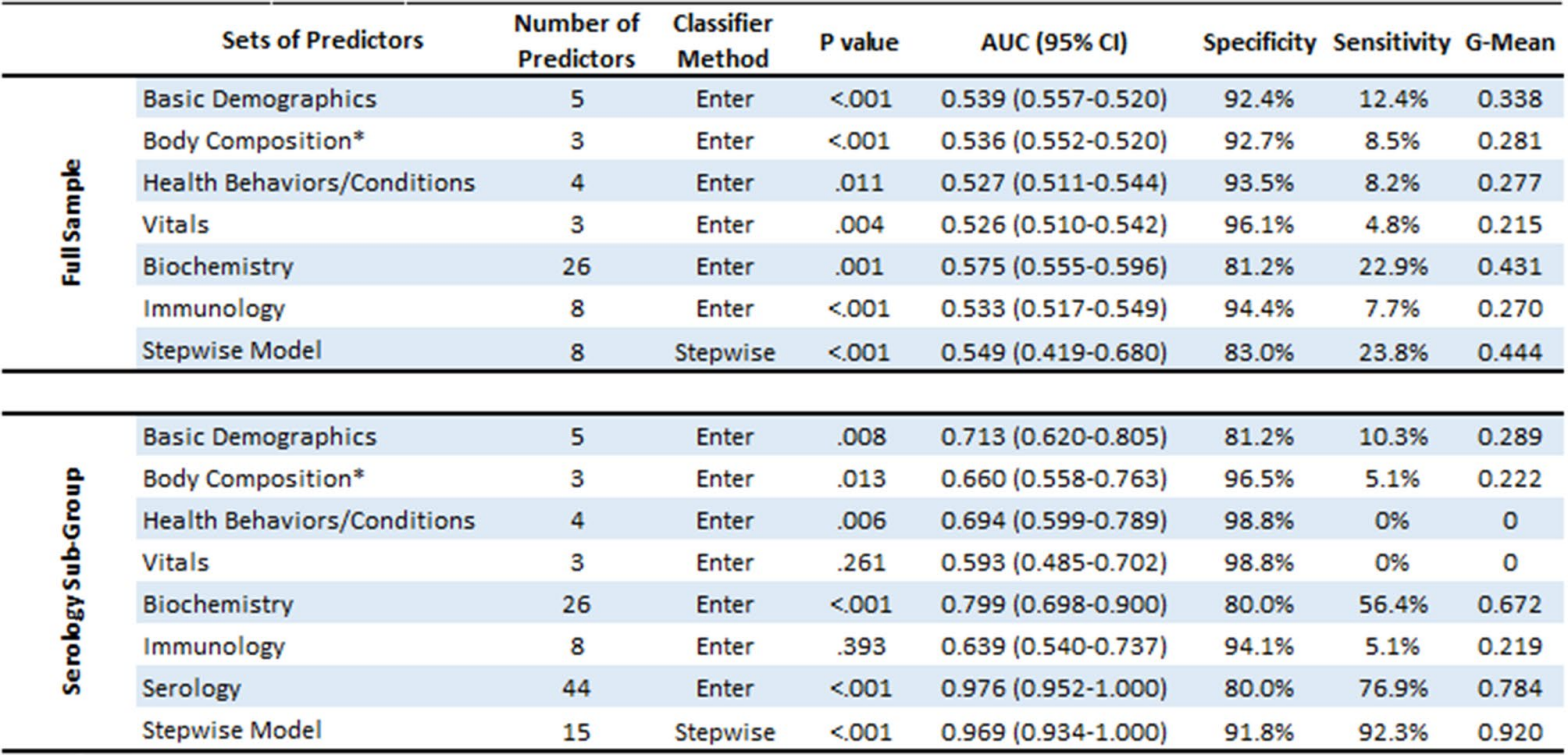
Sets of predictors used to predict classification of COVID-19 test cases as negative or positive. Area Under the Curve (AUC); Confidence Interval (CI); Geometric Mean (G-Mean). Non-parametric bootstrapping (1000 iterations, 95% CI) was used for robust estimation. P values less than 0.05 were considered significant. “bold” and “italics” shading are used to distinguish between predictors that loaded for a given model. ^a^Due to several variables representing the same construct (i.e., being multicollinear), body composition consisted of: whole-body water mass; whole-body fat mass; whole-body non-fat mass (i.e., muscle, bone).

Separately, another set of analyses determined how each baseline predictor could predict which of the 2210 positive COVID-19 cases had a mild or severe disease course. For context, 45% and 55% of test cases were mild or severe respectively. Among all positive test cases (Supplementary Table 5), significant predictors showed a trade-off between better sensitivity or specificity and were only modestly useful (AUC mean and range = 0.536, 0.524–0.572). Similarly, for the serology sub-group, Supplementary Table 6 shows that only alanine aminotransferase and neutrophil count significantly predicted disease severity. For serology data itself, Supplementary Table 7 indicates that the only significant predictors were U14 antigen to human herpesvirus 7 (AUC 0.729; g-mean = 0.600) and JC VP1 antigen to human JC polyomavirus (AUC 0.671; g-mean = 0.591). Table 4 shows the relative predictive value of groups of predictors for whether a COVID-19 infection would be severe. Figure 1 shows the ROC curves for model fit. Supplementary Table 8 illustrates that the stepwise model included only alanine aminotransferase, age in years, and monocyte count. For the serology sub-group, despite strong concerns about model overfitting, the AUC and g-mean were similarly modest compared to the full sample, except for the stepwise model that performed noticeably better (AUC 0.803; g-mean = 0.724). As shown in Supplementary Table 8, this model had only 2 predictors: antibody response to two antigens for two diseases (HTLV-1 gag for HTLV-1 and JC VP1 for Human Polyomavirus JCV).

**Table 4:**
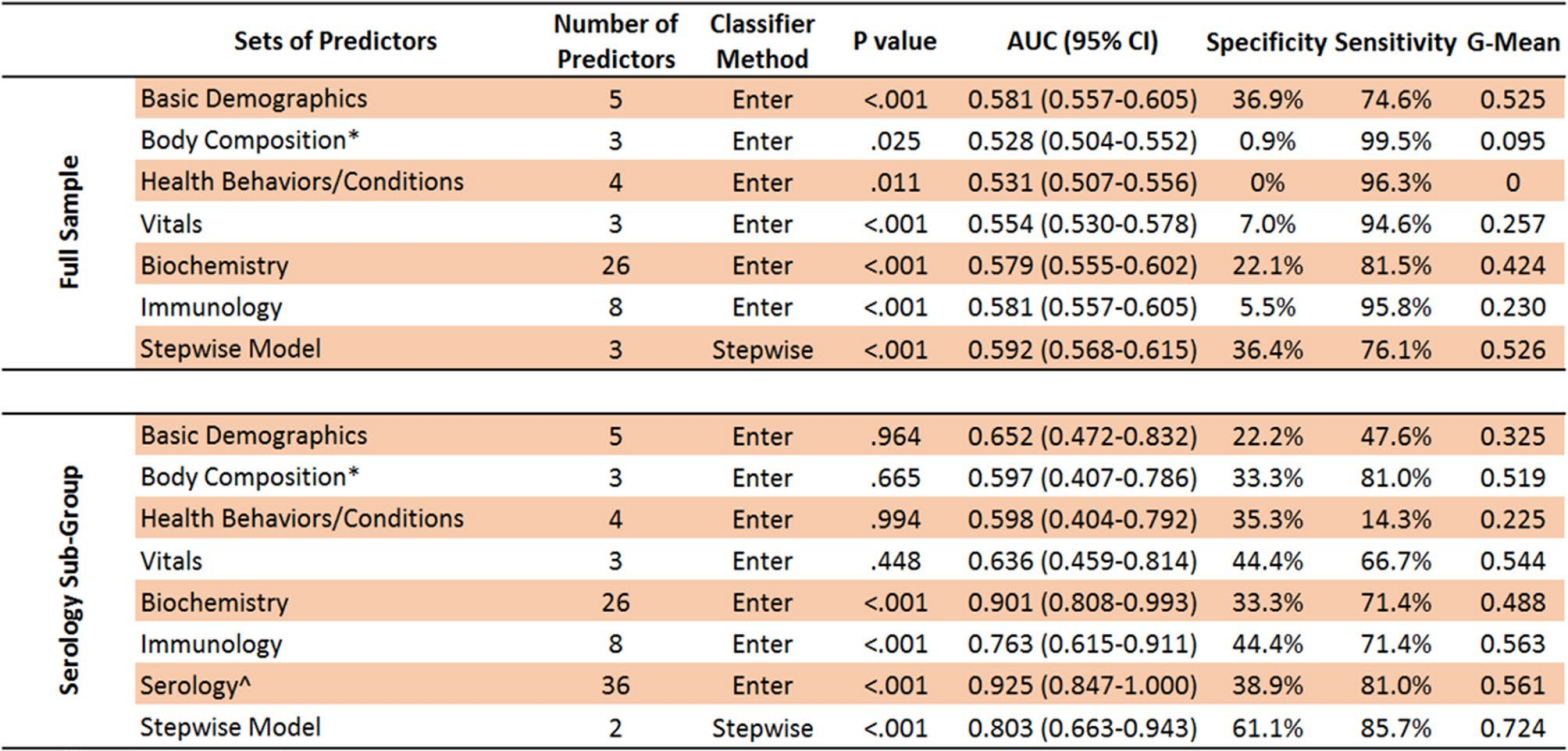
Sets of predictors used to predict classification of COVID-19 positive cases as mild or severe. Area Under the Curve (AUC); Confidence Interval (CI); Geometric Mean (G-Mean). Non-parametric bootstrapping (1000 iterations, 95% CI) was used for robust estimation. P values less than 0.05 were considered significant. “bold” and “italics” shading are used to distinguish between predictors that loaded for a given model. ^a^Due to several variables representing the same construct (i.e., being multicollinear), body composition consisted of: whole-body water mass; whole-body fat mass; whole-body non-fat mass (i.e., muscle, bone). ^b^Due to the full serology panel of 44 antibody titers exceeding degrees of freedom, titers for 6 antigens were excluded for pathogens with the lowest estimated prevalence in the cohort (HIV, HCV, HTLV-1).

## Discussion

The objectives of this study were to determine if baseline data from 2006 to 2010 could predict which older adults would develop COVID-19 in 2020, and if an infection was mild or presumptively severe due to being at hospital. In summary, using a permutation-based LDA approach, we developed separate risk profiles that did well at predicting test cases that were negative or positive (stepwise g-mean = 92%), and to some degree among positive test cases whether the infection was mild or severe (stepwise g-mean = 72.4%). Such profiles would require retrospective, routine self-report, blood test panels typically collected during annual medical wellness visits, and serology information for several antigens. As proof-of-principle that these profiles are sensible, we confirmed as others have noted that non-white ethnicity, low socioeconomic status, larger body mass, and alcohol use can increase infectious risk^5^.

Our most novel finding was that antibody titers to past infections were strong predictors of COVID-19 infection and severity, both as a group and especially in concert with established risk factors. This “virome” may consist of beneficial and detrimental pathogens, or fine-grained efficacy of the immune system to clear certain pathogens, that change how the immune system responds to a persistent viral challenge like COVID-19^10^. For example, antibodies to human cytomegalovirus antigens were the strongest predictors of infection risk in our stepwise model. Older adults with prior infection show exhaustion of the naïve T cell pool and fewer memory versus effector cells^27^. This may explain why monocyte count was one of the few variables to predict COVID-19 severity among all test cases in this study, as innate immunity must compensate for deficits in acquired immune function. For COVID-19 severity, antibody titers to the HTLV1 virus and human JC polyomavirus were the only predictors that loaded significantly in our stepwise model. While HTLV1 is rare, 57.5% of at least the UK Biobank sample have antibody levels that suggest prior infection with the human JC polyomavirus. This virus can induce hemagglutination in type O blood cells^28^, which may in some way influence why this blood type is protective against COVID-19 infection.

For other immunologic factors, as expected, mobilization of innate immunity was relevant to infection risk and severity. In particular, granulocytes (e.g., neutrophils, monocytes) loaded significantly in stepwise models for COVID-19 infection and severity, but not cytokines such as C-Reactive Protein. C-Reactive Protein has been cited as a strong risk factor for COVID-19^29^. However, this marker merely reflects signaling of the acute phase response due to systemic infection, and changes to granulocytes in circulation already reflect this response. Although lymphopenia and suppression of humoral immunity have been noted in COVID-19, lymphocyte cell count did not load in final stepwise models.

We also confirmed and extended the importance of age, lipids, vascular health, and socioeconomic status, but while body mass was important it was not adiposity per se. Among mostly elderly adults in our UK Biobank sample, age was one of the few factors to impact both infection and severity risk. Perhaps in concert, lipoprotein metabolism changes with aging can induce hyperlipidemia, which is a risk factor for cardiovascular disease and may increase COVID-19 infection risk^30^. The lack of association with anthropometric or bioimpedance-derived fat mass was unexpected, whereas fat-free mass such as muscle and bone did load as a factor. We speculate that more bone mineral density and somatic muscle would reflect less cardiometabolic impairment and systemic inflammation, but mechanisms are unclear. Finally, levels of testosterone weakly loaded as a predictive factor for who would later develop COVID-19. Sex differences favoring COVID-19 infection in men are clear, where andropause induces less testosterone production, which normally downregulates inflammation and could increase COVID-19 susceptibility^31^.

Several major limitations should be noted. The number of UK Biobank participants with COVID-19 and serology data is low, particularly for positive test cases. This could consequently lead to model overfitting or misestimation. Several steps were taken to guard against this problem, including feature reduction through LDA, bootstrapped parameter estimation to guard against parametric assumption violations, and several cross-validation steps to maximize robustness. We also rigorously tested each predictor or set of predictors in the main sample and serology sub-group, where we found that model fit was not overly biased in general despite sample size differences. Nonetheless, we recognize future work must use much larger sample sizes to verify the usefulness of serology data. Another limitation was that using test case data nested within a participant violates the assumption of independence, which can lead to gross misestimation. While we ameliorated this issue using permutation testing, other latent concerns with the data like type 2 error may be present. We also chose to use LDA over other machine learning algorithms, where LDA tends to provide more conservative estimates. This was intentional, because it is still largely unknown how risk factors alone or additively reflect overall risk for COVID-19 infection and disease severity. Finally, we only looked at the so called main effects of all predictors instead of complex interactions, such as darker skin, vitamin D levels, and COVID-19 infection risk. Such interactions were beyond the scope of this report, but may be promising avenues to explore in future studies.

## Conclusions

In summary, this study systematically used retrospective data in a community cohort to predict who would develop COVID-19 and if the disease course was presumptively severe. Despite baseline data having been collected 10–14 years ago, we achieved excellent to encouraging results by combining several sets of established and novel risk factors together. It is especially interesting that serological data performed as well as or better than other data types. Future work should leverage markers of host immunity to inform what may happen when the host is challenged by COVID-19.

## Supplementary Information

Supplementary Information.

## Data Availability

The data that support the findings of this study are available from the UK Biobank but restrictions apply to the availability of these data, which were used under license for the current study, and so are not publicly available. Data are however available from the authors upon reasonable request and with permission of UK Biobank. Permission is acquired through an application for data access to UK Biobank, https://www.ukbiobank.ac.uk/register-apply/.

## References

[1] Coronaviridae Study Group of the International Committee on Taxonomy of, V (2020). The species Severe acute respiratory syndrome-related coronavirus: Classifying 2019-nCoV and naming it SARS-CoV-2. Nat. Microbiol..

[2] Sattar N, McInnes IB, McMurray JJV (2020). Obesity a risk factor for severe COVID-19 infection: Multiple potential mechanisms. Circulation.

[3] Simonnet A (2020). High prevalence of obesity in severe acute respiratory syndrome coronavirus-2 (SARS-CoV-2) requiring invasive mechanical ventilation. Obesity (Silver Spring).

[4] Zhou F (2020). Clinical course and risk factors for mortality of adult inpatients with COVID-19 in Wuhan, China: A retrospective cohort study. Lancet.

[5] Patel AP, Paranjpe MD, Kathiresan NP, Rivas MA, Khera AV (2020). Race, socioeconomic deprivation, and hospitalization for COVID-19 in English participants of a National Biobank. medRxiv..

[6] Hamer M, Kivimaki M, Gale CR, David Batty G (2020). Lifestyle risk factors, inflammatory mechanisms, and COVID-19 hospitalization: A community-based cohort study of 387,109 adults in UK. Brain Behav. Immun..

[7] Liu Y (2020). Viral dynamics in mild and severe cases of COVID-19. Lancet Infect. Dis..

[8] Qin C (2020). Dysregulation of immune response in patients with COVID-19 in Wuhan, China. Clin. Infect. Dis..

[9] Li T (2004). Significant changes of peripheral T lymphocyte subsets in patients with severe acute respiratory syndrome. J. Infect. Dis..

[10] Moss P (2020). "The ancient and the new": Is there an interaction between cytomegalovirus and SARS-CoV-2 infection?. Immun. Ageing.

[11] Chidrawar S (2009). Cytomegalovirus-seropositivity has a profound influence on the magnitude of major lymphoid subsets within healthy individuals. Clin. Exp. Immunol..

[12] Sudlow C (2015). UK Biobank: An open access resource for identifying the causes of a wide range of complex diseases of middle and old age. PLoS Med..

[13] Armstrong, J. *et al.* Dynamic linkage of COVID-19 test results between Public Health England's Second Generation Surveillance System and UK Biobank. [Google Scholar]. (2020). 10.1099/mgen.0.000397 PMC7478634 32553051

[14] Hilton B (2020). Incidence of microbial infections in English UK Biobank participants: Comparison with the general population. medRxiv..

[15] Phillimore P, Beattie A, Townsend P (1994). Widening inequality of health in northern England, 1981–91. BMJ.

[16] Klinedinst BS (2019). Aging-related changes in fluid intelligence, muscle and adipose mass, and sex-specific immunologic mediation: A longitudinal UK Biobank study. Brain Behav. Immun..

[17] Kotler DP, Burastero S, Wang J, Pierson RN (1996). Prediction of body cell mass, fat-free mass, and total body water with bioelectrical impedance analysis: Effects of race, sex, and disease. Am. J. Clin. Nutr..

[18] Elliott P, Peakman TC (2008). The UK Biobank sample handling and storage protocol for the collection, processing and archiving of human blood and urine. Int. J. Epidemiol..

[19] Waterboer T, Sehr P, Pawlita M (2006). Suppression of non-specific binding in serological Luminex assays. J. Immunol. Methods.

[20] Armstrong J (2020). Dynamic linkage of COVID-19 test results between Public Health England's Second Generation Surveillance System and UK Biobank. Microb. Genom..

[21] Chadeau-Hyam M (2020). Risk factors for positive and negative COVID-19 tests: A cautious and in-depth analysis of UK biobank data. Int. J. Epidemiol..

[22] Hastie, T., Tibshirani, R. & Friedman, J. *The Elements of Statistical Learning: Data Mining, Inference, and Prediction*. (Springer Science & Business Media, 2009).

[23] Marron JS, Todd MJ, Ahn J (2007). Distance-weighted discrimination. J. Am. Stat. Assoc..

[24] Mundry R, Sommer C (2007). Discriminant function analysis with nonindependent data: Consequences and an alternative. Anim. Behav..

[25] Hair Jr, J. F., Anderson, R. E., Tatham, R. L. & Black, C. *Multivariate Data Analysis with Readings*. (Prentice Hall, 1995).

[26] Efron, B. *Breakthroughs in Statistics* 569–593 (Springer, 1992).

[27] Weinberger B (2007). Healthy aging and latent infection with CMV lead to distinct changes in CD8+ and CD4+ T-cell subsets in the elderly. Hum. Immunol..

[28] Osborn JE (1974). Comparison of JC and BK human papovaviruses with simian virus 40: Restriction endonuclease digestion and gel electrophoresis of resultant fragments. J. Virol..

[29] Liu W (2020). Analysis of factors associated with disease outcomes in hospitalized patients with 2019 novel coronavirus disease. Chin. Med. J. (Engl.).

[30] Wang D (2020). Clinical characteristics of 138 hospitalized patients with 2019 novel coronavirus-infected pneumonia in Wuhan, China. JAMA.

[31] Maggio M (2005). The relationship between testosterone and molecular markers of inflammation in older men. J. Endocrinol. Investig..

