# Supplementary material for: Using machine learning to predict COVID-19 infection and severity risk among 4510 aged adults: a UK Biobank cohort study
Source: Sci Rep. 2022 May 11;12:7736. doi: 10.1038/s41598-022-07307-z (PMC9092926; doi:10.1038/s41598-022-07307-z)
Supplement: Supplementary file 1 — Supplementary Information. [file 41598_2022_7307_MOESM1_ESM.pdf]

Title: Using machine learning to predict COVID-19 infection and severity risk among 4,510 aged adults: a UK Biobank cohort study

Auriel A. Willette, Ph.D.[1,2,3*]; Sara A. Willette, B.A.[3]; Qian Wang, Ph.D.[1]; Colleen Pappas, Ph.D.[1]; Brandon S. Klinedinst, M.S.[1]; Scott Le, B.S.[1]; Brittany Larsen, M.S.[1]; Amy Pollpeter, M.S.[1]; Tianqi Li, M.D.[1]; Jonathan P. Mochel[4]; Karin Allenspach[5]; Nicole Brenner, Ph.D.[6]; Tim Waterboer, Ph.D.[6]

(1) Department of Food Science and Human Nutrition, Iowa State University, Ames, IA, USA

(2) Department of Neurology, University of Iowa, Iowa City, IA, USA

(3) Iowa COVID-19 Tracker Inc., Ames, IA, USA

(4) Department of Biomedical Sciences, Iowa State University, Ames, IA, USA

(5) Department of Veterinary Clinical Sciences, Iowa State University, Ames, IA, USA

(6) Infections and Cancer Epidemiology Division, German Cancer Research Center (DKFZ), Heidelberg, Germany

[*]Address Correspondence to:
Auriel A. Willette, Ph.D., M.S.
2302 Osborn Drive
Ames, IA 50011-1078
Phone: (515) 294-3110
Email: Awillett@iastate.edu

Supplemental Text 1.

To reiterate, the main objectives of this report are to use Linear Discriminant Analysis (LDA) for probabilistic determination via naïve Bayesian classification of: 1) a two-class grouping defined as a positive vs. negative COVID-19 test case; and 2) a two-class grouping nested within positive COVID-19 tests, defined as a test case occurring in a hospital vs. non-hospital setting. For positive tests, these settings are considered as proxies for mild vs. severe COVID-19 disease status[1]. UK Biobank later confirmed disease severity using electronic health records and death certificates from pseudonymized Public Health England and other public records data. Because two or more test cases could be nested within a given participant, this would normally violate independence and potentially invalidate results. While one could use a single random test case per participant, this reduces sample size and does not represent real world data and within-subject variability (e.g., changes in COVID-19 status or infection severity). Thus, for estimation robust to non-independence, we used Mundry and Sommer's permuted LDA approach[2]. The unit of randomization across participants was a grouping of all test cases originally nested in a given participant. The null hypothesis was that a given LDA model with randomized data would not perform better than the original non-randomized data in 95% of permutations. As recommended, 1,000 permutations were run for each model using macros provided by the authors and Python scripting for automation. To ensure that models were stable and generalizable, we used a typical "holdout" method of 70% and 30% respectively for the training and test samples[3].

As discussed in the main text, forced entry models were first conducted for each predictor, and then among sets of similar predictors (e.g., demographics, vital signs). It was known from the outset that model overfitting would likely occur when a predictor set had dozens of features (e.g., biochemistry). This procedure was done for two reasons: 1) to show clinicians, researchers, and policymakers how a set of common features would discriminate COVID-19 groups in ideal circumstances, where in some cases model overfit is frankly likely; and 2) to contrast such models with the comparable or superior performance of stepwise models that had substantial feature reduction and enough $n > p$ that model overfit was guarded against.

We now explain why LDA was used. Foremost, prediction models derived using LDA are straightforward to interpret by a general audience, which is appropriate for the journal in question. For example, the Wilks' Lambda statistic allows a clear interpretation for how well a given predictor distinguishes between classes and its directionality (e.g., higher age in years predicts increased likelihood of positive COVID-19 classification). Equally important, LDA creates models that maximally separate classes of interest, where a new observation's data can be used to determine which class that observation would belong in. Since it is of central importance to have equally valid diagnostic assessment for who is and is not at risk for COVID-19 or if a positive would have a mild or severe infection, LDA is most appropriate. As a generative, supervised learning classification technique, LDA is also best used in complex datasets with high dimensionality composed of a few to hundreds of features per data category, where it can remove most redundant or dependent features that do not maximize model fit. Reducing the feature set size reduces the risk of model overfitting[4,5]. This procedure minimizes the High Dimensional, Low Sample Size (i.e., $p >> N$, or "small *n*, big *p*") problem[4] by reducing the likelihood of the within-class covariance matrix approaching singularity[6] and leading to instability of parameter estimation.

LDA has several key assumptions that we wish to address. LDA is relatively robust to overfitting provided there is a relative lack of outliers, multivariate normality and lack of multicollinearity, and independence of data values between participants. To begin, UK Biobank removes extreme values during data quality control before posting datasets to their data showcase[7]. We further log-transformed all quantitative variables to normalize distributions and "bring in" outliers defined as data points >3SD from the mean. As described in the main text, we also removed biochemistry variables that were multicollinear (e.g., direct vs. total bilirubin). While some antigens of the same pathogen approached multicollinearity, removing them from feature selection led to identical results and thus they were kept in. Participant-level data was not dependent on data from other participants. To be clear, however, multiple observations were nested within a given participant and would violate independence. Because the permutation LDA testing randomizes which participants have a group of one to several COVID-19 test cases, however, these models are robust to non-independence.

While other machine learning techniques are also appropriate for classification, we discuss why they were not used. Regarding logistic regression, this technique is attractive because it has no distribution assumptions. However, it assumes observations are independent. This does not occur with COVID-19 testing, in which a participant will often have multiple test cases. Logistic regression also requires a large numbers of observations to provide reliable estimates. Finally, it does not produce robust models for well-separate types of classes. As there are very clear immunologic differences that determine if someone has or does not have COVID-19, and a clear demarcation between mild vs. severe symptom presentation, we believe logistic regression model estimations might be inflated and thus less accurate. Finally, despite their methodological differences, LDA and logistic regression may perform the same with real data[8], where LDA may be more conservative and was one of our goals for this proof-of-principle study.

More complex algorithms vs. LDA were also not considered due to feature complexity, the need for transparent model estimates, and sample size. First, in the dataset there are many features present for biochemistry markers, antibody load to specific antigens, and to a lesser degree immune factors. Data reduction is therefore important to determine which features are most useful for COVID-19 data and should receive attention. By contrast, clustering methods are not suitable because the dimensionality space is too high and model fit is likely to be poor. For newer machine learning techniques, such as deep learning, it is often unclear what set of features are selected or their relative contribution when a given prediction is made. This is unacceptable for predicting COVID-19 infection or severity risk. For researchers, it is unknown how various risk factors converge to affect risk and this information is necessary to better understand underlying mechanisms. In population health or the clinic, certain features have prohibitive time or cost constraints (e.g., body compartment imaging; ordering one versus multiple antigen tests). More importantly, it is critical for clinicians, policy makers, or other stakeholders to point out which exact features led or would lead to a predicted outcome. Finally, deep learning, support vector machines (SVM), and similar approaches also require much larger sample sizes to train and adapt a classifier to produce robust estimates. By comparison, our dataset only had several thousand testing datapoints in the "full" sample and just over one-hundred in the sub-group that had serological data.

We recognize that LDA has several limitations and used non-parametric estimation to minimize these issues. To begin, using simulation data, LDA performs comparably to logistic regression when predictor distributions are normal or near normal, but has worse fit when there are clear normality violations[9]. While we log-

transformed quantitative measures with appreciable skewness (>3SD), normality nonetheless remained a concern, particularly for the serology sub-group that had 124 observations. To reduce potential problems, bootstrapping[10] was used (95% CI, 1000 iterations) to estimate model coefficients. This allows unbiased estimation of generalized absolute error, taking into account potential model overfit by substantially varying training and test sets from the selected sample. Nevertheless, with the serology sub-group, the small *n*, big *p* problem may still be a concern. Regularized LDA has been a popular choice to overcome this issue of within-class covariance singularity, where cross-validation presents a reasonable solution[11]. Due to computation problems in tandem with bootstrapping, we used a simple “leave-one-out” approach with bootstrap estimates.

Supplementary Text 2.

This section addresses why test case data for COVID-19 test was used, and to what degree participants had within-subject variability for testing data. Typically, all test cases are examined to determine if a given person was ever positive for COVID-19, and if infected whether that infection was mild or required hospitalization (i.e., severe). This data is then used to create binary categorical outcomes of risk. While this approach is straightforward, it may lead to misestimation and bias that limits the generalizability of results.

First, for adults tested more than once for COVID-19 in the UK, there are meaningful differences in key risk factors. As shown by Chadeau-Hyam and colleagues[12] in supplemental material, participants with multiple tests (40.5%) were more likely to have lower education attainment, lower average household income and greater economic hardship (e.g., renting vs. owning, unemployment %, etc.), and a substantially greater likelihood of having comorbid conditions. This was not due to occupation bias it seems, as participants with multiple tests were less likely to be a frontline healthcare worker (15.46% to 9.04%). In effect, ignoring these differences may give rise to sampling bias. This bias can lead to gross over- or underestimation of the "true" resilience or risk conferred by a predictor variable, in the same way that there is frequent misestimation of COVID-19 prevalence[13]. By examining all test cases and using LDA-based permutation testing[14] to ensure robustness of model estimates at the test case level, we have a more meaningful idea of how a given person reacts to COVID-19 exposures over time. In this way, such models: 1) are better able to control for non-random sampling that will occur during a worldwide pandemic; and 2) are more robust and generalizable to "real-world" conditions, in which out-patients and particularly in-patients are likely to have more than one COVID-19 test done.

Collider bias is also a concern, which is highlighted by Griffith et al. using UK Biobank data[15]. Briefly, a collider is a variable influenced by a given risk factor and an outcome. For COVID-19, the authors indicate this bias occurs when restricting analyses to a subset of participants that ever developed COVID-19 or were hospitalized (i.e., had severe disease). This sampling may distort patterns of association and limit generalizability to the general population. Further, it can distort "individual-level causal effects." As an example, between March to late April 2020, symptomatic emergency responders and health-care workers were more frequently tested in hospital settings than community members, whereas testing policy changed after late April to more broadly address community testing. Collider variables here would be occupation (e.g., healthcare worker), degree of exposure, and at what date/dates a given sample was taken. While Griffith et al. go on to discuss collider bias in tested vs. non-tested UK Biobank participants, we argue that "throwing out" all but one test case per participant introduces collider bias. Specifically, this would lead to artificially overweighting participants who had 1 test case and underweighting participants who had multiple test cases and are more likely to be one of several disadvantaged groups prone to COVID-19 infection and cases of severe infection[12].

While using all test case data can mitigate these issues to some degree, a new problem that occurs is within-subject variability. We operationally define this variability as a participant who, based on a new COVID-19 test instance, "switches" groups for either the Disease Risk variable (negative; positive) or the Severity Risk variable (mild; severe). To test the effect of within-subject variability, we used Chadeau-Hyam et al.'s approach[12] to categorize all UK Biobank participants into a three-level categorical variable, Testing Frequency:

- Adults who had one COVID-19 test
- Adults who had two COVID-19 tests
- Adults who had three or more COVID-19 tests

Using a linear mixed effects model, we compared the "2 test" group vs. the "1 test" group, and then the "3 or more tests" group with the "2 test group." We first tested disease risk using the 'Result' variable from UK Biobank. We next assessed severity risk using the 'Origin' variable from UK Biobank. Critically, if a given test case's result was negative (i.e., Result=0), that test case was not included in the severity risk analysis. Put in other words, because there is no disease present and therefore no affiliated symptoms, it does not matter if a negative test was done in an outpatient or inpatient setting. We emphasize that this is the prescribed methodology[1] and has been used in numerous published UK Biobank studies on COVID-19 risk.

For participants with 2 tests, there was a 2% higher likelihood for the second test to be COVID-19 positive (32.3% to 34.3%, p<.001) vs. the first test. Similarly, there was a 6% higher likelihood for the second test of a COVID-19+ patient to reflect severe instead of mild symptoms (55.2% to 61.4%, p<.001). For participants with 3 or more tests, there was no higher likelihood of being COVID-19 positive (34.3% to 34.3%, p=0.988) vs. the second test. Curiously, for participants in the 3 or more test group that tested positive for COVID-19, there was a 3.7% lower likelihood of having severe instead of mild symptoms (65.1% vs. 61.4%). Finally, there were no significant interactions to suggest that number of tests or different Testing Frequency groups influenced sensitivity or specificity. Likewise, model estimates for positivity or severity did not change when introducing number of tests or Testing Frequency as a covariate.

These results suggest that repeated testing reflects a significant but modest increase in the likelihood that a COVID-19 test will be positive. For severity risk, our results suggest that the change across testing groups reflects the standard of care for hospitals at that time[1]. Specifically, from March to mid-April 2020 and to a lesser extent mid-April to mid-June 2020, symptomatic COVID-19 participants would be initially tested at hospital for COVID-19, admitted to hospital if positive, up to a week later have a 2nd test done (often a PCR) to confirm positivity and check for viral load, and up to a week later potentially be tested again. In many cases, for patients who clearly still had severe symptoms, a 3rd test was not done. A 3rd test would be more likely to occur if symptoms improved for the patient and there was a possibility for discharge.

Supplemental References

1 Armstrong, J. *et al.* Dynamic linkage of COVID-19 test results between Public Health England's Second Generation Surveillance System and UK Biobank. *Microbial genomics* **6**, doi:10.1099/mgen.0.000397 (2020).

2 Mundry, R. & Sommer, C. Discriminant function analysis with nonindependent data: consequences and an alternative. *Animal Behaviour* **74**, 965-976 (2007).

3 Hair Jr, J. F., Anderson, R. E., Tatham, R. L. & Black, C. *Multivariate data analysis with readings*. (Prentice Hall, 1995).

4 Hastie, T., Tibshirani, R. & Friedman, J. *The elements of statistical learning: data mining, inference, and prediction*. (Springer Science & Business Media, 2009).

5 Marron, J. S., Todd, M. J. & Ahn, J. Distance-weighted discrimination. *Journal of the American Statistical Association* **102**, 1267-1271 (2007).

6 Qiao, Z., Zhou, L. & Huang, J. Z. Sparse Linear Discriminant Analysis with Applications to High Dimensional Low Sample Size Data. *International Journal of Applied Mathematics* **39** (2009).

7 Sudlow, C. *et al.* UK Biobank: An open access resource for identifying the causes of a wide range of complex diseases of middle and old age. *PLOS Medicine* **12**, e1001779, doi:10.1371/journal.pmed.1001779 (2015).

8 Rausch, J. R. & Kelley, K. A comparison of linear and mixture models for discriminant analysis under nonnormality. *Behavior Research Methods* **41**, 85-98 (2009).

9 Pohar, M., Blas, M. & Turk, S. Comparison of logistic regression and linear discriminant analysis: a simulation study. *Metodoloski zvezki* **1**, 143 (2004).

10 Efron, B. in *Breakthroughs in statistics*, 569-593 (Springer, 1992).

11 Ye, J. *et al.* in *Proceedings of the 15th ACM international conference on Information and knowledge management.* 532-539.

12 Chadeau-Hyam, M. *et al.* Risk factors for positive and negative COVID-19 tests: a cautious and in-depth analysis of UK biobank data. *International journal of epidemiology* **49**, 1454-1467, doi:10.1093/ije/dyaa134 (2020).

13 Díaz-Pachón, D. A. & Rao, J. S. A simple correction for COVID-19 sampling bias. *Journal of theoretical biology* **512**, 110556, doi:10.1016/j.jtbi.2020.110556 (2021).

14 Mundry, R. & Sommer, C. Discriminant function analysis with nonindependent data: consequences and an alternative. *Animal Behaviour* **74**, 965-976 (2007).

15 Griffith, G. J. *et al.* Collider bias undermines our understanding of COVID-19 disease risk and severity. *Nature communications* **11**, 5749, doi:10.1038/s41467-020-19478-2 (2020).

Supplementary Table 1. Isolated effect of each non-serology predictor on COVID-19 risk among the full sample

Predictor	Classifier Method	P value	AUC (95% CI)	Specificity	Sensitivity	G-Mean
**Basic Demographics**						
**Age**	**Enter**	**0.002**	**0.522 (0.505-0.538)**	**86.9%**	**17.3%**	**0.388**
**Sex**	**Enter**	**0.033**	**0.517 (0.501-0.534)**	**100%**	**0%**	**0**
Ethnic Background	Enter	0.080	0.514 (0.498-0.531)	93.6%	11.2%	0.324
**Deprivation Index**	**Enter**	**<.001**	**0.540 (0.524-0.556)**	**79.5%**	**22.5%**	**0.423**
Education	Enter	0.626	0.505 (0.479-0.512)	100%	0%	0
**Body Composition**						
**Waist Circumference**	**Enter**	**<.001**	**0.544 (0.528-0.561)**	**97.0%**	**2.7%**	**0.162**
**Body Mass Index**	**Enter**	**<.001**	**0.548 (0.531-0.564)**	**94.0%**	**6.4%**	**0.245**
**Trunk Fat Mass**	**Enter**	**<.001**	**0.533 (0.516-0.549)**	**97.4%**	**3.3%**	**0.179**
**Whole Body Fat Mass**	**Enter**	**0.002**	**0.526 (0.509-0.542)**	**91.1%**	**8.8%**	**0.283**
**Whole Body Fat-Free Mass**	**Enter**	**0.005**	**0.529 (0.513-0.545)**	**91.4%**	**9.6%**	**0.296**
**Whole Body Water Mass**	**Enter**	**0.005**	**0.529 (0.512-0.545)**	**91.5%**	**9.6%**	**0.296**
**Health Behaviors and Conditions**						
Smoking Status	Enter	0.167	0.511 (0.472-0.505)	100%	0%	0
**Alcohol Status**	**Enter**	**<.001**	**0.517 (0.501-0.533)**	**88.6%**	**14.6%**	**0.360**
Long-Term Medical Condition	Enter	0.691	0.504 (0.488-0.520)	100%	0%	0
Health Rating	Enter	0.354	0.503 (0.481-0.514)	100%	0%	0
**Vitals**						
Pulse Rate	Enter	0.954	0.503 (0.481-0.514)	100%	0%	0
**Diastolic BP**	**Enter**	**0.041**	**0.519 (0.503-0.536)**	**97.3%**	**3.3%**	**0.179**
Systolic BP	Enter	0.952	0.501 (0.483-0.516)	100%	0%	0
**Biochemistry**						
Alanine Aminotransferase	Enter	0.302	0.512 (0.491-0.533)	99.8%	0%	0
Albumin	Enter	0.154	0.504 (0.484-0.525)	100%	0%	0
Alkaline Phosphatase	Enter	0.781	0.508 (0.487-0.528)	100%	0%	0
**Apolipoprotein A**	**Enter**	**<.001**	**0.536 (0.515-0.557)**	**96.4%**	**3.0%**	**0.170**
Apolipoprotein B	Enter	0.192	0.514 (0.493-0.534)	99.8%	0.1%	0.032
Aspartate Aminotransferase	Enter	0.284	0.518 (0.498-0.539)	100%	0%	0
Bilirubin (Total)	Enter	0.377	0.501 (0.480-0.521)	100%	0.1%	0.032
Calcium	Enter	0.104	0.517 (0.497-0.538)	99.8%	0.4%	0.063
Cholesterol (Total)	Enter	0.833	0.517 (0.484-0.525)	100%	0%	0
Creatinine	Enter	0.898	0.505 (0.515-0.556)	100%	0%	0
**Cystatin C**	**Enter**	**0.032**	**0.523 (0.502-0.543)**	**100%**	**0%**	**0**
Gamma Glutamyltransferase	Enter	0.648	0.502 (0.481-0.522)	100%	0%	0
**HDL Cholesterol**	**Enter**	**<.001**	**0.536 (0.515-0.557)**	**95.2%**	**4.3%**	**0.202**
**Hemoglobin A1c**	**Enter**	**0.006**	**0.537 (0.516-0.558)**	**98.3%**	**1.8%**	**0.133**
Insulin-Like Growth Factor 1	Enter	0.316	0.504 (0.483-0.525)	100%	0%	0
LDL Cholesterol	Enter	0.105	0.504 (0.484-0.525)	100%	0%	0
Lipoprotein A	Enter	0.081	0.503 (0.482-0.524)	100%	0%	0

Phosphate	Enter	0.173	0.515 (0.494-0.536)	100%	0%	0
Protein (Total)	Enter	0.078	0.517 (0.497-0.538)	100%	0%	0
**Sex Hormone Binding Globulin**	**Enter**	**<.001**	**0.551 (0.531-0.572)**	**100%**	**0%**	**0**
Testosterone	Enter	0.096	0.513 (0.492-0.533)	100%	0%	0
Triglycerides	Enter	0.098	0.517 (0.497-0.538)	97.7%	2.7%	0.162
Urate	Enter	0.105	0.517 (0.496-0.538)	98.9%	1.3%	0.113
Urea	Enter	0.081	0.504 (0.484-0.525)	100%	0%	0
Vitamin D	Enter	0.512	0.501 (0.480-0.521)	100%	0%	0
**Immunology**						
**Red Blood Cell Count**	**Enter**	**0.001**	**0.524 (0.509-0.539)**	**90.7%**	**11.6%**	**0.324**
White Blood Cell Count	Enter	0.426	0.505 (0.490-0.520)	100%	0.2%	0.045
C-Reactive Protein	Enter	0.394	0.511 (0.496-0.525)	99.2%	0.9%	0.094
Neutrophils	Enter	0.071	0.521 (0.507-0.536)	99.4%	0.7%	0.083
Lymphocytes	Enter	0.053	0.518 (0.503-0.533)	99.8%	0.3%	0.055
Monocytes	Enter	0.172	0.505 (0.490-0.519)	98.6%	1.6%	0.126
Eosinophils	Enter	0.853	0.514 (0.499-0.528)	100%	0%	0
Basophils	Enter	0.086	0.510 (0.495-0.525)	100%	0%	0

Area Under the Curve (AUC); Confidence Interval (CI); Geometric Mean (G-Mean). Specificity and sensitivity are the likelihood of correctly detecting when COVID-19 infection for a test case was negative or positive respectively. G-Mean is the degree to which a given predictor correctly predicts both true negatives and true positives for COVID-19 infection. “Blue” and “white” shading are used to better visualize predictors within a set of similar variables. P values less than .05 were considered significant and applicable predictors and statistics are bolded.

Supplementary Table 2. Isolated effect of each predictor on COVID-19 risk among test cases with serology data

Predictor	Classifier Method	P value	AUC (95% CI)	Specificity	Sensitivity	G-Mean
**Basic Demographics**						
Age	Enter	0.634	0.568 (0.441-0.694)	100%	0%	0
**Sex**	**Enter**	**<.001**	**0.689 (0.578-0.800)**	**66.0%**	**71.8%**	**0.688**
Ethnic Background	Enter	0.647	0.528 (0.407-0.649)	98.1%	12.8%	0.354
Deprivation Index	Enter	0.704	0.539 (0.416-0.662)	100%	0%	0
**Education**	**Enter**	**0.017**	**0.635 (0.522-0.748)**	**64.2%**	**53.8%**	**0.588**
**Body Composition**						
Waist Circumference	Enter	0.142	0.598 (0.480-0.716)	83.0%	10.3%	0.292
Body Mass Index	Enter	0.575	0.548 (0.427-0.669)	96.2%	0%	0
Trunk Fat Mass	Enter	0.792	0.539 (0.413-0.665)	100%	0%	0
Whole Body Fat Mass	Enter	0.252	0.582 (0.464-0.701)	92.5%	5.1%	0.217
**Whole Body Fat-Free Mass**	**Enter**	**0.003**	**0.687 (0.575-0.799)**	**71.7%**	**59.0%**	**0.650**
**Whole Body Water Mass**	**Enter**	**0.003**	**0.680 (0.567-0.793)**	**71.7%**	**59.0%**	**0.650**
**Health Behaviors and Conditions**						
Smoking Status	Enter	0.072	0.610 (0.494-0.726)	47.2%	0%	0
Alcohol Status	Enter	0.094	0.603 (0.482-0.723)	100%	20.5%	0.453
Long-Term Medical Condition	Enter	0.391	0.546 (0.427-0.666)	100%	0%	0
Health Rating	Enter	0.661	0.521 (0.403-0.639)	100%	0%	0
**Vitals**						
Pulse Rate	Enter	0.335	0.582 (0.461-0.702)	92.5%	2.6%	0.155
**Diastolic BP**	**Enter**	**0.047**	**0.633 (0.515-0.752)**	**84.9%**	**35.9%**	**0.552**
Systolic BP	Enter	0.200	0.540 (0.420-0.660)	83.0%	12.8%	0.326
**Biochemistry**						
Alanine Aminotransferase	Enter	0.303	0.588 (0.463-0.714)	100%	0%	0
Albumin	Enter	0.285	0.676 (0.525-0.828)	93.0%	0%	0
Alkaline Phosphatase	Enter	0.272	0.541 (0.422-0.660)	98.1%	0%	0
Apolipoprotein A	Enter	0.333	0.600 (0.463-0.737)	95.3%	0%	0
Apolipoprotein B	Enter	0.125	0.545 (0.426-0.665)	88.7%	15.4%	0.370
Aspartate Aminotransferase	Enter	0.399	0.520 (0.398-0.641)	96.2%	0%	0
Bilirubin (Total)	Enter	0.319	0.543 (0.420-0.665)	88.7%	0%	0
Calcium	Enter	0.989	0.528 (0.386-0.670)	100%	0%	0
Cholesterol (Total)	Enter	0.276	0.528 (0.408-0.649)	90.6%	5.1%	0.215
Creatinine	Enter	0.250	0.524 (0.403-0.646)	88.7%	5.1%	0.213
Cystatin C	Enter	0.169	0.634 (0.512-0.755)	88.7%	7.7%	0.261
Gamma Glutamyltransferase	Enter	0.103	0.604 (0.482-0.725)	100%	0%	0
HDL Cholesterol	Enter	0.640	0.551 (0.410-0.692)	100%	0%	0
**Hemoglobin A1c**	**Enter**	**0.035**	**0.638 (0.523-0.753)**	**90.6%**	**30.8%**	**0.528**
Insulin-Like Growth Factor 1	Enter	0.852	0.533 (0.407-0.659)	100%	0%	0
LDL Cholesterol	Enter	0.247	0.533 (0.413-0.654)	84.9%	5.1%	0.208
Lipoprotein A	Enter	0.460	0.554 (0.418-0.691)	95.8%	0%	0

**Phosphate**	**Enter**	**0.026**	**0.671 (0.531-0.811)**	**88.4%**	**4.3%**	**0.195**
**Protein (Total)**	**Enter**	**0.014**	**0.662 (0.514-0.811)**	**90.7%**	**47.8%**	**0.658**
Sex Hormone Binding Globulin	Enter	0.900	0.510 (0.360-0.659)	100%	0%	0
**Testosterone**	**Enter**	**0.021**	**0.731 (0.625-0.836)**	**86.8%**	**35.9%**	**0.558**
Triglycerides	Enter	0.063	0.614 (0.498-0.729)	71.7%	71.8%	0.717
Urate	Enter	0.088	0.656 (0.538-0.774)	86.8%	20.5%	0.422
**Urea**	**Enter**	**0.021**	**0.632 (0.508-0.757)**	**86.8%**	**35.9%**	**0.558**
**Vitamin D**	**Enter**	**0.009**	**0.601 (0.479-0.722)**	**81.1%**	**38.5%**	**0.559**
**Immunology**						
C-Reactive Protein	Enter	0.394	0.567 (0.447-0.687)	99.2%	0.9%	0.094
Red Blood Cell Count	Enter	0.943	0.504 (0.384-0.625)	90.7%	11.6%	0.324
White Blood Cell Count	Enter	0.426	0.592 (0.476-0.709)	100%	0.2%	0.045
**Neutrophils**	**Enter**	**0.037**	**0.628 (0.507-0.749)**	**99.4%**	**0.7%**	**0.083**
Lymphocytes	Enter	0.053	0.552 (0.434-0.671)	99.8%	0.3%	0.055
**Monocytes**	**Enter**	**0.020**	**0.646 (0.532-0.761)**	**98.6%**	**1.6%**	**0.126**
**Eosinophils**	**Enter**	**0.015**	**0.649 (0.535-0.762)**	**100%**	**0%**	**0**
Basophils	Enter	0.086	0.528 (0.409-0.647)	100%	0%	0

Area Under the Curve (AUC); Confidence Interval (CI); Geometric Mean (G-Mean). Specificity and sensitivity are the likelihood of correctly detecting when COVID-19 infection for a test case was negative or positive respectively. G-Mean is the degree to which a given predictor correctly predicts both true negatives and true positives for COVID-19 infection. “Blue” and “white” shading are used to better visualize predictors within a set of similar variables. P values less than .05 were considered significant and applicable predictors and statistics are bolded.

Supplementary Table 3. Isolated effect of each baseline antibody titer on predicting current COVID-19 infection risk

Pathogen Name	Abbreviation	Antigen	Classifier Method	P value	AUC (95% CI)	Specificity	Sensitivity	G-Mean
Herpes Simplex Virus-1	HSV-1	1gG	Enter	0.089	0.526 (0.407-0.645)	56.3%	51.3%	0.537
Herpes Simplex Virus-2	HSV-2	2mgG	Enter	0.422	0.518 (0.402-0.634)	18.8%	82.1%	0.393
Varicella Zoster Virus	VZV	**gE/gl**	**Enter**	**0.046**	**0.612 (0.509-0.714)**	**31.3%**	**69.2%**	**0.465**
Epstein-Barr Virus	EBV	**VCA p18**	**Enter**	**0.002**	**0.675 (0.574-0.775)**	**0%**	**100.0%**	**0**
		EBNA-1	Enter	0.996	0.549 (0.441-0.658)	0%	94.9%	0
		ZEBRA	Enter	0.419	0.589 (0.49-0.688)	0%	100.0%	0
		EA-D	Enter	0.815	0.503 (0.397-0.609)	0%	89.7%	0
Human Cytomegalovirus	CMV	**pp150 Nter**	**Enter**	**0.006**	**0.654 (0.539-0.769)**	**50.0%**	**74.4%**	**0.610**
		pp 52	Enter	0.285	0.555 (0.442-0.668)	43.8%	74.4%	0.571
		pp 28	Enter	0.284	0.602 (0.485-0.718)	34.4%	74.4%	0.506
Human Herpesvirus-6	HHV-6	IE1A	Enter	0.110	0.586 (0.476-0.697)	31.3%	76.9%	0.491
		**IE1B**	**Enter**	**0.007**	**0.638 (0.539-0.738)**	**37.5%**	**82.1%**	**0.555**
		p101 k	Enter	0.687	0.532 (0.431-0.633)	0%	92.3%	0
Human Herpesvirus-7	HHV-7	**U14**	**Enter**	**0.003**	**0.684 (0.577-0.792)**	**65.6%**	**66.7%**	**0.661**
Kaposi's Sarcoma Associated Herpesvirus	KSHV	LANA	Enter	0.899	0.546 (0.439-0.653)	0%	94.9%	0
		K8.1	Enter	0.912	0.564 (0.452-0.675)	0%	94.9%	0
Hepatitus B Virus	HBV	HBc	Enter	0.808	0.505 (0.399-0.611)	0%	100.0%	0
		HBe	Enter	0.434	0.600 (0.486-0.713)	3.1%	82.1%	0.160
Hepatitus C Virus	HCV	Core	Enter	0.627	0.524 (0.415-0.633)	0%	94.9%	0
		**NS3**	**Enter**	**0.011**	**0.663 (0.559-0.766)**	**50.0%**	**71.8%**	**0.599**
Toxoplasma gondii	T. gondii	**p22**	**Enter**	**0.036**	**0.617 (0.517-0.718)**	**40.6%**	**84.6%**	**0.586**
		**sag1**	**Enter**	**0.038**	**0.662 (0.554-0.769)**	**37.5%**	**82.1%**	**0.555**
Human T Lymphotropic Virus 1	HTLV-1	**HTLV-1 gag**	**Enter**	**0.003**	**0.710 (0.618-0.802)**	**56.3%**	**82.1%**	**0.680**
		HTLV-1 env	Enter	0.402	0.554 (0.451-0.658)	18.8%	92.3%	0.417
Human Immunodeficiency Virus	HIV	**HIV-1 gag**	**Enter**	**0.001**	**0.688 (0.592-0.785)**	**37.5%**	**84.6%**	**0.563**
		HIV-1 env	Enter	0.204	0.577 (0.469-0.685)	31.3%	69.2%	0.465
Human Polyomavirus BKV	BKV	**BK VP1**	**Enter**	**0.008**	**0.649 (0.539-0.759)**	**53.1%**	**59.0%**	**0.560**
Human Polyomavirus JCV	JCV	JC VP1	Enter	0.796	0.530 (0.419-0.641)	0%	100.0%	0
Merkel Cell Polyomavirus	MCV	MC VP1	Enter	0.648	0.546 (0.442-0.649)	0%	89.7%	0
	HPV 16	L1	Enter	0.961	0.525 (0.412-0.637)	0%	100.0%	0
		**E6**	**Enter**	**0.029**	**0.622 (0.517-0.728)**	**53.1%**	**66.7%**	**0.595**

Human Papillomavirus type-16		**E7**	**Enter**	**0.009**	**0.646 (0.536-0.756)**	**34.4%**	**74.4%**	**0.506**
Human Papillomavirus type-18	HPV 18	L1	Enter	0.404	0.556 (0.453-0.660)	12.5%	84.6%	0.325
Chlamydia trachomatis	C. trachomatis	momp D	Enter	0.455	0.501 (0.390-0.612)	3.1%	97.4%	0.174
		momp A	Enter	0.075	0.551 (0.450-0.653)	28.1%	89.7%	0.502
		tarp-D F1	Enter	0.918	0.549 (0.431-0.668)	0%	100.0%	0
		tarp-D F2	Enter	0.814	0.572 (0.459-0.686)	0%	100.0%	0
		PorB	Enter	0.352	0.597 (0.488-0.706)	9.4%	87.2%	0.286
		**pGP3**	**Enter**	**0.005**	**0.656 (0.547-0.765)**	**21.9%**	**79.5%**	**0.417**
Helicobacter pylori	H. pylori	CagA*	N/A	N/A	N/A	N/A	N/A	N/A
		**VacA**	**Enter**	**0.045**	**0.613 (0.506-0.719)**	**25.0%**	**87.2%**	**0.467**
		OMP	Enter	0.770	0.509 (0.397-0.622)	0%	94.9%	0
		GroEL	Enter	0.308	0.591 (0.467-0.715)	28.1%	74.4%	0.457
		Catalase	Enter	0.663	0.525 (0.416-0.634)	0%	89.7%	0
		UreA	Enter	0.290	0.567 (0.461-0.672)	18.8%	87.2%	0.405

Area Under the Curve (AUC); Confidence Interval (CI); Geometric Mean (G-Mean). Specificity and sensitivity are the likelihood of correctly detecting if COVID-19 infection for a test case was negative or positive respectively. G-Mean is the degree to which a given antigen correctly predicts both true negatives and true positives for COVID-19 infection. “Blue” and “white” shading are used to better visualize antigens specific to a given pathogen. P values less than .05 were considered significant and applicable antigens and statistics are bolded. *The CagA antigen was excluded from analysis due to roughly half of sample analyte values being lost to lab error.

Supplementary Table 4. Predictors that loaded into the stepwise models for COVID-19 infection risk

	Stepwise Predictor	Wilks' λ	Coefficient	Seroprevalence
**All Test Cases**	Ethnicity	0.983	-0.319	
	Triglycerides	0.983	0.355	
	Townsend Deprivation Index	0.983	0.417	
	Age in Years	0.983	0.26	
	Monocyte Count	0.984	-0.246	
	Whole Body Fat-Free Mass	0.984	0.231	
	Alcohol Status	0.984	0.377	
	Diastolic Blood Pressure	0.985	-0.292	
**Serology Sub-Group**	pp 52 antigen for Human Cytomegalovirus	0.332	0.507	58.2%
	Gamma Glutamyltransferase	0.334	0.268	
	Erythrocyte Count	0.334	-0.339	
	PorB Antigen for Chlamydia trachomatis	0.336	-0.404	21.4%
	Cholesterol	0.339	-0.353	
	Triglycerides	0.341	-0.369	
	pp 28 Antigen for Human Cytomegalovirus	0.345	-0.754	58.2%
	IE1A Antigen for Human Herpesvirus 6	0.356	-0.490	90.8%
	Monocyte Count	0.382	-0.651	
	Age in Years	0.384	0.656	
	pGP3 Antigen for Chlamydia trachomatis	0.408	0.731	21.4%
	Neutrophil Count	0.420	0.782	
	NS3 Antigen for Hepatitis C Virus	0.423	0.804	0.3%
	Urate	0.469	-0.961	
	Testosterone	0.608	1.441	

Wilks' λ represents the relative strength of a given predictor in contributing to the final model fit. Seroprevalence is the proportion of participants whose assay values were high enough such that they were considered positive for having a given disease. "Blue" and "white" shading are used to distinguish between predictors that loaded for a given model.

Supplementary Table 5. Isolated effect of each predictor on COVID-19 severity among the full sample

Predictor	Classifier Method	P value	AUC (95% CI)	Specificity	Sensitivity	G-Mean
**Basic Demographics**						
**Age**	**Enter**	**<.001**	**0.572 (0.548-0.596)**	**75.5%**	**36.7%**	**0.526**
**Sex**	**Enter**	**0.009**	**0.528 (0.504-0.552)**	**100%**	**0%**	**0**
**Ethnic Background**	**Enter**	**<.001**	**0.524 (0.500-0.548)**	**91.0%**	**12.9%**	**0.343**
Deprivation Index	Enter	0.610	0.507 (0.483-0.531)	91.0%	12.9%	0.343
Education	Enter	0.854	0.505 (0.478-0.532)	100%	0%	0
**Body Composition**						
**Waist Circumference**	**Enter**	**0.003**	**0.541 (0.517-0.565)**	**93.6%**	**6.0%**	**0.237**
Body Mass Index	Enter	0.198	0.522 (0.498-0.547)	100%	0%	0
Trunk Fat Mass	Enter	0.068	0.531 (0.506-0.555)	99.9%	0.1%	0.032
Whole Body Fat Mass	Enter	0.341	0.521 (0.497-0.546)	100%	0%	0
Whole Body Fat-Free Mass	Enter	0.104	0.522 (0.498-0.547)	100%	0%	0
Whole Body Water Mass	Enter	0.095	0.522 (0.497-0.546)	100%	0%	0
**Health Behaviors and Conditions**						
Smoking Status	Enter	0.114	0.522 (0.497-0.546)	100%	0%	0
Alcohol Status	Enter	0.540	0.506 (0.482-0.530)	100%	0%	0
Long-Term Medical Condition	Enter	0.084	0.519 (0.494-0.543)	100%	0%	0
Health Rating	Enter	0.098	0.518 (0.494-0.543)	100%	0%	0
**Vitals**						
Pulse Rate	Enter	0.652	0.510 (0.486-0.535)	100%	0%	0
Diastolic BP	Enter	0.969	0.501 (0.476-0.526)	100%	0%	0
**Systolic BP**	**Enter**	**0.008**	**0.540 (0.515-0.565)**	**98.0%**	**2.0%**	**0.140**
**Biochemistry**						
**Alanine Aminotransferase**	**Enter**	**0.039**	**0.540 (0.515-0.565)**	**100%**	**0%**	**0**
Albumin	Enter	0.093	0.525 (0.498-0.552)	98.9%	0.4%	0.063
Alkaline Phosphatase	Enter	0.198	0.522 (0.496-0.547)	100%	0%	0
Apolipoprotein A	Enter	0.245	0.513 (0.487-0.540)	99.7%	0.1%	0.032
Apolipoprotein B	Enter	0.862	0.502 (0.477-0.528)	100%	0%	0
**Aspartate Aminotransferase**	**Enter**	**0.047**	**0.534 (0.508-0.559)**	**100%**	**0%**	**0**
Bilirubin (Total)	Enter	0.965	0.509 (0.483-0.534)	100%	0%	0
Calcium	Enter	0.545	0.507 (0.481-0.534)	99.8%	0%	0
Cholesterol (Total)	Enter	0.352	0.510 (0.484-0.535)	100%	0%	0
Creatinine	Enter	0.289	0.510 (0.485-0.535)	100%	0%	0
**Cystatin C**	**Enter**	**0.006**	**0.527 (0.502-0.553)**	**99.5%**	**0.7%**	**0.083**
Gamma Glutamyltransferase	Enter	0.181	0.529 (0.504-0.555)	100%	0%	0
HDL Cholesterol	Enter	0.180	0.517 (0.490-0.544)	98.7%	0.4%	0.063
**Hemoglobin A1c**	**Enter**	**0.002**	**0.555 (0.529-0.580)**	**99.3%**	**0.7%**	**0.083**
**Insulin-Like Growth Factor 1**	**Enter**	**0.037**	**0.524 (0.499-0.550)**	**96.8%**	**3.8%**	**0.192**
LDL Cholesterol	Enter	0.470	0.508 (0.483-0.533)	100%	0%	0

Lipoprotein A	Enter	0.216	0.518 (0.489-0.546)	100%	0%	0
Phosphate	Enter	0.357	0.513 (0.486-0.540)	99.9%	0%	0
Protein (Total)	Enter	0.930	0.512 (0.486-0.539)	100%	0%	0
**Sex Hormone Binding Globulin**	**Enter**	**0.036**	**0.525 (0.498-0.552)**	**94.2%**	**7.5%**	**0.266**
Testosterone	Enter	0.060	0.521 (0.495-0.548)	100%	0%	0
Triglycerides	Enter	0.060	0.528 (0.503-0.554)	100%	0%	0
**Urate**	**Enter**	**0.012**	**0.533 (0.508-0.559)**	**98.5%**	**1.7%**	**0.129**
**Urea**	**Enter**	**0.003**	**0.530 (0.505-0.556)**	**97.8%**	**2.7%**	**0.162**
Vitamin D	Enter	0.562	0.503 (0.477-0.529)	100%	0%	0
**Immunology**						
Red Blood Cell Count	Enter	0.732	0.504 (0.479-0.529)	100%	0%	0
**White Blood Cell Count**	**Enter**	**0.025**	**0.536 (0.511-0.561)**	**100%**	**0%**	**0**
C-Reactive Protein	Enter	0.598	0.528 (0.503-0.554)	100%	0%	0
**Neutrophils**	**Enter**	**0.004**	**0.535 (0.510-0.560)**	**98.2%**	**3.6%**	**0.188**
Lymphocytes	Enter	0.212	0.504 (0.479-0.530)	100%	0%	0
Monocytes	Enter	0.071	0.530 (0.505-0.555)	100%	0%	0
Eosinophils	Enter	0.291	0.520 (0.495-0.545)	100%	0%	0
Basophils	Enter	0.671	0.500 (0.475-0.525)	100%	0%	0

Area Under the Curve (AUC); Confidence Interval (CI); Geometric Mean (G-Mean). Here, specificity and sensitivity are the likelihood of correctly detecting if a positive COVID-19 test case was mild or severe respectively. G-Mean is the degree to which a given predictor correctly predicts both true negatives and true positives for COVID-19 infection severity. "Orange" and "white" shading are used to better visualize each class of predictors for COVID-19 severity. P values less than .05 were considered significant, where applicable predictors and classifier statistics are bolded.

Supplementary Table 6. Isolated effect of each predictor on COVID-19 severity for the serology sub-group

Predictor	Classifier Method	P value	AUC (95% CI)	Specificity	Sensitivity	G-Mean
**Basic Demographics**						
Age	Enter	0.889	0.532 (0.348-0.716)	81.0%	0%	0
Sex	Enter	0.455	0.556 (0.373-0.738)	33.3%	0%	0
Ethnic Background	Enter	0.889	0.520 (0.331-0.708)	95.2%	0%	0
Deprivation Index	Enter	0.973	0.520 (0.331-0.708)	85.7%	0%	0
Education	Enter	0.706	0.504 (0.296-0.712)	100%	0%	0
**Body Composition**						
Waist Circumference	Enter	0.308	0.612 (0.430-0.795)	85.7%	27.8%	0.488
Body Mass Index	Enter	0.363	0.517 (0.328-0.706)	81.0%	22.2%	0.424
Trunk Fat Mass	Enter	0.087	0.615 (0.423-0.806)	75.0%	47.1%	0.594
Whole Body Fat Mass	Enter	0.100	0.629 (0.437-0.822)	90.0%	35.3%	0.564
Whole Body Fat-Free Mass	Enter	0.763	0.515 (0.325-0.704)	100%	0%	0
Whole Body Water Mass	Enter	0.851	0.562 (0.372-0.752)	100%	0%	0
**Health Behaviors and Conditions**						
Smoking Status	Enter	0.798	0.516 (0.331-0.701)	71.4%	0%	0
Alcohol Status	Enter	0.845	0.505 (0.321-0.689)	100%	0%	0
Long-Term Medical Condition	Enter	0.802	0.521 (0.334-0.708)	100%	0%	0
Health Rating	Enter	0.999	0.501 (0.317-0.686)	100%	0%	0
**Vitals**						
Pulse Rate	Enter	0.128	0.593 (0.412-0.773)	71.4%	33.3%	0.488
Diastolic BP	Enter	0.984	0.520 (0.334-0.705)	90.5%	0%	0
Systolic BP	Enter	0.868	0.513 (0.324-0.702)	85.7%	0%	0
**Biochemistry**						
**Alanine Aminotransferase**	**Enter**	**0.043**	**0.690 (0.511-0.870)**	**47.6%**	**77.8%**	**0.609**
Albumin	Enter	0.483	0.579 (0.332-0.827)	85.7%	0%	0
Alkaline Phosphatase	Enter	0.311	0.538 (0.350-0.727)	71.4%	33.3%	0.488
Apolipoprotein A	Enter	0.892	0.587 (0.351-0.824)	92.9%	0%	0
Apolipoprotein B	Enter	0.587	0.542 (0.358-0.727)	85.7%	0%	0
Aspartate Aminotransferase	Enter	0.688	0.642 (0.463-0.820)	95.2%	0%	0
Bilirubin (Total)	Enter	0.482	0.586 (0.404-0.768)	57.1%	22.2%	0.356
Calcium	Enter	0.190	0.635 (0.406-0.864)	85.7%	22.0%	0.434
Cholesterol (Total)	Enter	0.945	0.517 (0.333-0.701)	81.0%	0%	0
Creatinine	Enter	0.096	0.649 (0.474-0.825)	76.2%	55.6%	0.651
Cystatin C	Enter	0.497	0.545 (0.355-0.735)	81.0%	27.8%	0.475
Gamma Glutamyltransferase	Enter	0.992	0.577 (0.393-0.760)	100%	0%	0
HDL Cholesterol	Enter	0.841	0.540 (0.286-0.794)	85.7%	0%	0
Hemoglobin A1c	Enter	0.506	0.560 (0.368-0.752)	65.0%	29.4%	0.437
Insulin-Like Growth Factor 1	Enter	0.786	0.544 (0.354-0.734)	95.2%	0%	0
LDL Cholesterol	Enter	0.808	0.546 (0.362-0.731)	85.7%	0%	0
Lipoprotein A	Enter	0.287	0.607 (0.385-0.829)	46.2%	71.4%	0.574

Phosphate	Enter	0.627	0.524 (0.278-0.770)	100%	0%	0
Protein (Total)	Enter	0.513	0.571 (0.335-0.808)	100%	0%	0
Sex Hormone Binding Globulin	Enter	0.578	0.587 (0.347-0.828)	100%	0%	0
Testosterone	Enter	0.723	0.634 (0.446-0.821)	100%	0%	0
Triglycerides	Enter	0.989	0.540 (0.351-0.728)	90.5%	0%	0
Urate	Enter	0.372	0.597 (0.413-0.779)	71.4%	33.3%	0.488
Urea	Enter	0.300	0.604 (0.419-0.790)	76.2%	44.4%	0.582
Vitamin D	Enter	0.877	0.500 (0.315-0.685)	100%	0%	0
**Immunology**						
Red Blood Cell Count	Enter	0.970	0.553 (0.361-0.746)	95.2%	0%	0
White Blood Cell Count	Enter	0.177	0.646 (0.455-0.836)	61.9%	35.3%	0.467
C-Reactive Protein	Enter	0.234	0.522 (0.322-0.723)	100%	22.2%	0.471
**Neutrophils**	**Enter**	**0.049**	**0.653 (0.475-0.830)**	**61.9%**	**52.9%**	**0.572**
Lymphocytes	Enter	0.581	0.548 (0.358-0.737)	95.2%	0%	0
Monocytes	Enter	0.822	0.534 (0.343-0.724)	90.5%	0%	0
Eosinophils	Enter	0.694	0.516 (0.323-0.709)	100%	0%	0
Basophils	Enter	0.153	0.604 (0.423-0.785)	76.2%	35.3%	0.519

Area Under the Curve (AUC); Confidence Interval (CI); Geometric Mean (G-Mean). Here, specificity and sensitivity are the likelihood of correctly detecting if a positive COVID-19 test case was mild or severe respectively. G-Mean is the degree to which a given predictor correctly predicts both true negatives and true positives for COVID-19 infection severity. "Orange" and "white" shading are used to better visualize each set of predictors for COVID-19 severity. P values less than .05 were considered significant, where applicable predictors and statistics are bolded.

Supplementary Table 7. Isolated effect of each baseline antibody titer on predicting current COVID-19 infection severity

Pathogen Name	Abbreviation	Antigen	Classifier Method	P value	AUC (95% CI)	Specificity	Sensitivity	G-Mean
Herpes Simplex Virus-1	HSV-1	1gG	Enter	0.185	0.626 (0.447-0.804)	61.1%	57.1%	0.591
Herpes Simplex Virus-2	HSV-2	2mgG	Enter	0.625	0.511 (0.321-0.701)	0%	90.5%	0
Varicella Zoster Virus	VZV	gE/gl	Enter	0.220	0.594 (0.412-0.776)	38.9%	61.9%	0.491
Epstein-Barr Virus	EBV	VCA p18	Enter	0.686	0.565 (0.381-0.748)	0%	81.0%	0
		EBNA-1	Enter	0.087	0.634 (0.452-0.815)	33.3%	81.0%	0.519
		ZEBRA	Enter	0.221	0.604 (0.421-0.788)	38.9%	71.4%	0.527
		EA-D	Enter	0.285	0.599 (0.418-0.780)	44.4%	61.9%	0.524
Human Cytomegalovirus	CMV	pp150 Nter	Enter	0.465	0.585 (0.399-0.771)	44.4%	71.4%	0.563
		pp 52	Enter	0.649	0.512 (0.322-0.702)	0%	81.0%	0
		pp 28	Enter	0.763	0.544 (0.355-0.733)	0%	90.5%	0
Human Herpesvirus-6	HHV-6	IE1A	Enter	0.592	0.538 (0.354-0.723)	0%	85.7%	0
		IE1B	Enter	0.700	0.565 (0.375-0.755)	0%	95.2%	0
		p101 k	Enter	0.667	0.507 (0.319-0.694)	0%	90.5%	0
Human Herpesvirus-7	HHV-7	U14	Enter	**0.016**	**0.729 (0.568-0.890)**	**44.4%**	**81.0%**	**0.600**
Kaposi's Sarcoma Associated Herpesvirus	KSHV	LANA	Enter	1.000	0.616 (0.437-0.796)	0%	95.2%	0
		K8.1	Enter	0.785	0.560 (0.371-0.748)	0%	95.2%	0
Hepatitus B Virus	HBV	HBc	Enter	0.850	0.587 (0.402-0.773)	0%	95.2%	0
		HBe	Enter	0.736	0.583 (0.350-0.727)	0%	95.2%	0
Hepatitus C Virus	HCV	Core	Enter	0.314	0.503 (0.316-0.689)	11.1%	100%	0.333
		NS3	Enter	0.847	0.578 (0.395-0.762)	0%	100%	0
Toxoplasma gondii	T. gondii	p22	Enter	0.259	0.549 (0.357-0.741)	5.6%	100%	0.237
		sag1	Enter	0.229	0.565 (0.379-0.751)	27.8%	90.5%	0.502
Human T Lymphotropic Virus 1	HTLV-1	HTLV-1 gag	Enter	0.065	0.647 (0.469-0.825)	66.7%	66.7%	0.667
		HTLV-1 env	Enter	0.570	0.595 (0.414-0.776)	11.1%	81.0%	0.300
Human Immunodeficiency Virus	HIV	HIV-1 gag	Enter	0.364	0.538 (0.353-0.724)	16.7%	85.7%	0.378
		HIV-1 env	Enter	0.634	0.534 (0.349-0.720)	0%	90.5%	0
Human Polyomavirus BKV	BKV	BK VP1	Enter	0.782	0.562 (0.380-0.744)	0%	81.0%	0
Human Polyomavirus JCV	JCV	JC VP1	Enter	**0.045**	**0.671 (0.502-0.840)**	**66.7%**	**52.4%**	**0.591**
Merkel Cell Polyomavirus	MCV	MC VP1	Enter	0.294	0.628 (0.448-0.809)	55.6%	61.9%	0.587
Human Papillomavirus type-16	HPV 16	L1	Enter	0.554	0.525 (0.338-0.712)	11.1%	81.0%	0.300
		E6	Enter	0.740	0.538 (0.349-0.728)	0%	95.2%	0
		E7	Enter	0.134	0.565 (0.382-0.748)	72.2%	52.4%	0.615

Human Papillomavirus type-18	HPV 18	L1	Enter	0.828	0.511 (0.322-0.699)	0%	85.7%	0
Chlamydia trachomatis	C. trachomatis	momp D	Enter	0.818	0.511 (0.322-0.699)	0%	95.2%	0
		momp A	Enter	0.819	0.505 (0.315-0.695)	0%	95.2%	0
		tarp-D F1	Enter	0.809	0.585 (0.403-0.766)	0%	95.2%	0
		tarp-D F2	Enter	0.615	0.538 (0.343-0.734)	0%	90.5%	0
		PorB	Enter	0.832	0.504 (0.319-0.689)	0%	95.2%	0
		pGP3	Enter	0.464	0.603 (0.422-0.784)	11.1%	95.2%	0.325
Helicobacter pylori	H. pylori	CagA*	N/A	N/A	NA	N/A	N/A	N/A
		VacA	Enter	0.915	0.602 (0.420-0.784)	0%	85.7%	0
		OMP	Enter	0.340	0.558 (0.375-0.741)	0%	47.6%	0
		GroEL	Enter	0.415	0.614 (0.433-0.795)	22.2%	85.7%	0.436
		Catalase	Enter	0.335	0.642 (0.464-0.819)	16.7%	95.2%	0.399
		UreA	Enter	0.300	0.606 (0.425-0.786)	0%	100%	0

Area Under the Curve (AUC); Confidence Interval (CI); Geometric Mean (G-Mean). Here, specificity and sensitivity are the likelihood of correctly detecting if a positive COVID-19 test case was mild or severe respectively. G-Mean is the degree to which a given antigen correctly predicts both true negatives and true positives for COVID-19 infection severity. "Orange" and "white" shading are used to better visualize each set of antigens for a specific pathogen. P values less than .05 were considered significant, where applicable antigens and statistics are bolded. *The CagA antigen was excluded from analysis due to roughly half of sample analyte values being lost to lab error.

Supplementary Table 8. Predictors that loaded into the stepwise models for COVID-19 severity risk

	Stepwise Predictor	Wilks' λ	Coefficient	Seroprevalence
**All Test Cases**	Alanine Aminotransferase	0.979	0.298	
	Age in Years	0.994	0.873	
	Monocyte Count	0.980	0.351	
**Serology Sub-Group**	HTLV-1 gag for Human T Lymphotropic Virus 1	0.896	0.926	1.6%
	JC VP1 antigen for Human Polyomavirus JCV	0.911	0.959	57.5%

Wilks' λ represents the relative strength of a given predictor in contributing to the final model fit. Seroprevalence is the proportion of participants whose assay values were high enough such that they were considered positive for having a given disease. "Orange" and "white" shading are used to better visualize each predictor that loaded into a given model.
